# Challenges of Introgression in Conservation: Genetic Diversity of the Endangered Wild Camel (*Camelus ferus*) in Mongolia

**DOI:** 10.1002/ece3.73293

**Published:** 2026-03-29

**Authors:** Anna M. Jemmett, John G. Ewen, Adiya Yadamsuren, Deborah A. Dawson, Lucy Raggett, Pamela A. Burger, Jim. J. Groombridge

**Affiliations:** ^1^ Durrell Institute of Conservation and Ecology, School of Natural University of Kent Canterbury UK; ^2^ Wild Camel Protection Foundation London UK; ^3^ Institute of Zoology Zoological Society of London London UK; ^4^ NERC Environmental Omics Facility‐Visitor Facility, School of Biosciences University of Sheffield, Western Bank Sheffield UK; ^5^ Wild Camel Protection Foundation Mongolia Ulaan Baatar Mongolia; ^6^ Research Institute of Wildlife Ecology University of Veterinary Medicine Vienna Austria

**Keywords:** *Camelus bacterianus*, *Camelus ferus*, conservation genetics, Great Gobi A Strictly Protected Area, hybridization, introgression, non‐invasive sampling

## Abstract

The endangered Wild Camel ( *Camelus ferus* Mongolian: хавтгай, *khavtgai*; Chinese: 野骆驼, *ye luo tuo*) is the only extant wild species of the *Camelini* tribe. Surviving only in remote areas of the Gobi deserts of Mongolia and China, this species is threatened with extinction through climate change, associated habitat disruptions and small population impacts. The Wild Camel can hybridize with domestic Bactrian Camel ( *Camelus bactrianus* ), and although this introgression has been considered a threat to species survival, the extent to which it has occurred is unknown. DNA was extracted from 257 individuals, from predominantly non‐invasive samples, collected across the in situ and ex situ Wild Camel populations in Mongolia. Genotyping with nuclear markers combined with mitochondrial DNA sequencing was used to gain a greater understanding of the extent and source of introgression and levels of genetic diversity in these populations. Results show evidence of nuclear, mitochondrial, and historic introgression of Bactrian Camel genes in the Wild Camel population in situ, and in some Wild Camel individuals within the ex situ herd. Nuclear introgression was detected between 10% and 22% of the in situ population in Mongolia. Mitochondrial and nuclear DNA analysis have allowed for the sources of introgression to be understood, with paternal introgression being the major source in both in situ and ex situ populations. Results have also shown reduced heterozygosity and elevated inbreeding in the in situ population and reveal similar characteristics in the ex situ herd. Although hybridization is often considered a threat, it may also be an opportunity for species' population viability, and this dilemma creates challenges in conservation management. Whilst the global conservation community currently adopts largely arbitrary thresholds for what is an acceptable level of introgression, a detailed genetic perspective is crucial in understanding hybridization and its effect on conservation.

## Graphical abstract

Using non‐invasive nuclear and mitochondrial analysis, this study reveals evidence of introgression in critically endangered Wild Camels ( *Camelus ferus* ) from the domestic Bactrian camel ( *Camelus bactrianus* ), alongside reduced genetic diversity and higher inbreeding in both in situ and ex situ populations in Mongolia. These findings highlight the need for detailed genetic assessments to guide conservation decisions, as current thresholds for acceptable introgression remain largely arbitrary.

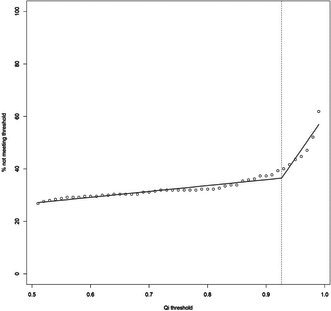

## 1 Introduction

**FIGURE 1:**
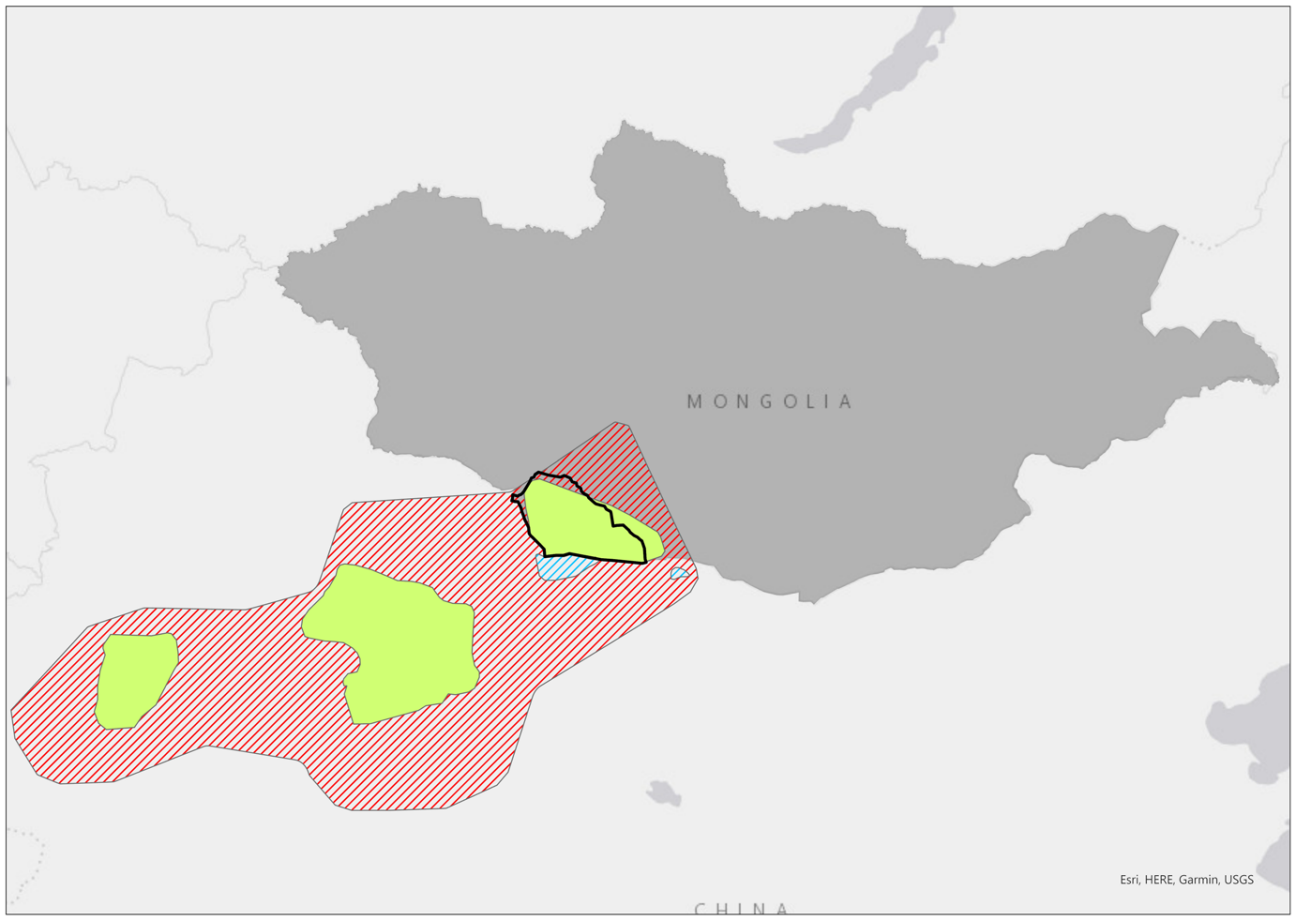
Wild Camel ( *C. ferus* ) range. Extant/resident (Green), Extinct (red) and possibly extinct (blue). Extent of the Great Gobi A Strictly Protected Area is outlined in black. Range update May 2025 IUCN Redlist.

The endangered (IUCN RedList 2026) Wild Camel, *Camelus ferus* (Mongolian: хавтгай, *khavtgai*; Chinese: 野骆驼, *ye luo tuo*) is the last remaining truly wild species of *Camelini* (Burger et al. 2019). The wild population (estimated 950 animals; IUCN RedList 2026) is restricted to four fragmented populations in the Gobi desert (Figure 1): three in northwest China (Burger 2016) (Taklamakan desert, Gashun Gobi Desert and Arjin Mountains in the Lop Nur Lake region (Lei et al. 2012); and one in Mongolia: the Great Gobi A Special Protected Area (GGASPA); IUCN RedList 2026). The GGASPA is a nature reserve that covers 53,000 km^2^ of Gobi Desert ecosystem. A single ex situ population of 34 individuals (at time of sample collection in April 2021) is managed by the Wild Camel Protection Foundation (WCPF) in Mongolia (Jemmett 2024) in the buffer zone of the GGASPA. As the only ex situ population of this species, they are held within the native range as an insurance against extinction in the wild.

The Gobi is one of the worlds least human influenced ecosystems (McCarthy et al. 2022). A key biodiversity site, this healthy desert habitat supports rare, endangered, and endemic species adapted to surviving extreme conditions, such as the Wild Camel. The rarity and global importance of the Gobi is reflected in its nomination for UNESCO World Heritage status (McCarthy et al. 2022). But climate change is causing increasing desertification of the Gobi (Han et al. 2021) making it the fastest growing desert worldwide (McCarthy et al. 2022). For the Wild Camel climate change is already predicted to cause of loss of large areas of suitable habitat (Xue et al. 2021). Loss of habitat and drying of water points may also increase contact between wild and domestic species, increasing not only risk disease and predation but also the risk of hybridization.

The Wild Camel (described throughout as *C. ferus* ) is a separate species to the globally common Bactrian Camel (described throughout as *C. bactrianus* ), having diverged from a common ancestor approximately 1.1 million years ago (MYA) (Mohandesan et al. 2017), but the two species can hybridize. *C. bactrianus* , a domestic species, is abundant, with over 450,000 individuals in Mongolia alone (Jemmett et al. 2023). Due to the close relatedness of the two species, hybrid offspring are viable and back‐crossing is possible, leaving *C. ferus* at risk of exposure to genetic swamping (Senn et al. 2019). In cases like the wild cat in Scotland, which is considered a hybrid swarm (Senn et al. 2019), effective conservation management can be even more complex. In Mongolia, introgression from *C. bactrianus* has previously been documented in the *C. ferus* population (Burger et al. 2019) using mitochondrial (Silbermayr et al. 2010), nuclear (Burger 2016) and Y chromosomal DNA (Felkel et al. 2019), but its population‐wide extent and conservation implications have remained unknown.

Within a conservation context, hybridization is challenging. Hybridization has the capacity to conserve unique genetic diversity by increasing heterozygosity and contributing to heterosis, thereby enhancing overall population fitness (Draper et al. 2021; Taylor and Larson 2019), but in highly threatened species, endemic genetic diversity may already be reduced and hybridization could reduce diversity further by risking loss of locally adapted or specialized traits and potentially contributing to population extinction (Rhymer and Simberloff 1996; Todesco et al. 2016). Genetic diversity underpins adaptive potential in populations, which is increasingly important in many systems undergoing rapid environmental change. Threatened populations are often small, isolated, genetically impoverished or inbred, and therefore improving future population trajectory with other genetic conspecifics is not always possible; in these instances, hybridization might be considered beneficial, as a form of genetic rescue (Vedder et al. 2022). As well as using hybrids to increase diversity, hybrids themselves can be conserved as a reservoir for the genetic material of a threatened parental species (as has been proposed in grey wolf/dog admixture; Pilot et al. 2018) or for performing a necessary ecological function (Steiner et al. 2017). It may also now be possible to use ex situ breeding programmes and whole genome sequencing to identify and remove hybrid segments of DNA from populations (Lawson et al. 2023). Hybridization can therefore either help or hinder conservation efforts depending on the context, meaning that managing hybridization is rarely straightforward.

Detection of hybrids can also be difficult. Until the 1960s, hybrids were identified through morphological phenotypes alone (Allendorf et al. 2001), but hybrids can express a range of phenotypes that are not necessarily intermediate between parent species, especially after several generations of backcrossing (Rhymer and Simberloff 1996). Furthermore, phenotypes of hybrids are not easily predictable: they can display morphological characteristics intermediate between parents (Fukami et al. 2019); alternatively, hybrids can be cryptic (Jasińska et al. 2010); or they may express phenotypes unlike either parent (Eliason et al. 2023). Therefore, phenotype can be a poor predictor of hybridization, which creates further challenges for conservation, as has been shown for the Scottish wild cat, for which pelage scores alone are insufficient, requiring molecular techniques for more accurate assessment (Senn et al. 2019).

Genetic analysis allows for the monitoring of hybridization across populations through time and space regardless of phenotype and is an important tool for managing populations of threatened species. There are a variety of methods available to monitor introgression, using both nuclear (Senn et al. 2019) and mitochondrial DNA (Silbermayr et al. 2010). However, using molecular data is also not straightforward. The extent of introgression within a population varies across a hybrid spectrum; crucially, there is no consensus as to what is an acceptable individual level of genetic admixture that does not compromise the evolutionary integrity of a species (Allendorf et al. 2001). Much of this uncertainty is because hybridization occurs most often between closely related species and thus the reliability of any comparison is dictated by the quality of the reference genome, the markers used and presence of genotyping errors (Ottenburghs 2021). Furthermore, evolutionarily distant hybrid ancestry can be difficult to detect with traditional methods (Taylor and Larson 2019) such as phenotype or fragment analysis. Modern advancement of whole genome sequence analysis may allow for improved quantification of benefit or threat of hybridization to species conservation (Van Oosterhout et al. 2022) or reveal further genomic complexity of hybridization and the evolutionary histories of species (Yuan et al. 2014).

In Mongolia, *C. ferus* and *C. bactrianus* exemplify the challenge of managing hybridization in conservation, one is a range‐restricted, highly adapted, critically endangered species on the brink of extinction; the other is a globally distributed domestic species. Although there are morphological differences between the two species, hybrids are often cryptic, and the small population size of *C. ferus* and remoteness of its habitat means that identifying hybrids phenotypically is difficult. In this study, non‐invasive sampling was applied across the Great Gobi A Strictly Protected Area (GGASPA) (Kaczensky et al. 2014), and a combined suite of nuclear and mitochondrial DNA markers were used to: (i) determine extent and source of nuclear introgression in both the ex situ and in situ Mongolian populations; (ii) evaluate the prevalence of this introgression based on hybrid threshold estimates currently adopted by the conservation community; (iii) identify hybridization patterns in mitochondrial DNA and compare them to patterns of nuclear introgression to identify drivers of hybridization; and (iv) quantify and interpret within the context of introgression, population genetic diversity and structure across the in situ population distributed throughout the GGASPA and the ex situ herd in Mongolia.

## 2 Research Methodology

### 2.1 Sample Collection

In situ samples were collected during two sample collection field trips in September 2018 and September 2019 in the GGASPA national park in Mongolia (Jemmett 2024). Samples were collected across the GGASPA (*n* = 262), with GPS locations recorded for each sample. To ensure minimal disturbance to wildlife and the ecosystem, a non‐invasive sampling protocol was employed. Samples collected included feces, hair caught in vegetation, and tissue taken from carcasses and bones from dead *C. ferus* found in the GGASPA. These samples were naturally dried by desert conditions and were stored and transported dried with silica beads.

Ex situ tissue samples were collected by a registered veterinarian from all 34 ex situ born *C. ferus* in October 2022 (permission granted by the Wild Camel Protection Foundation; WCPF 2023) during standard veterinary ear‐tagging procedures. Ear tagging procedures used DALTON flexo‐DNA ear tags (Dalton 2026) which stored the sample before transportation. At the time of sample collection in April 2022, all individuals from the ex situ population were successfully sampled from.

Fecal samples were collected from 5 known domestic *C. bactrianus* in herder‐inhabited areas surrounding the GGASPA. These samples were naturally dried by desert conditions and were stored and transported dried with silica beads.

Combined with DNA extractions from samples previously collected from known *C. bactrianus* , *C. ferus* and hybrid Mongolian and Chinese camels, *n* = 89 (Silbermayr et al. 2010) and genotype data, *n* = 59 (Silbermayr and Burger 2012), the final sample set comprised a total of 449 samples.

### 2.2 Laboratory Analysis

Depending on sample type, DNA was extracted using either a QIAGEN DNeasy blood and tissue kit (tissue, blood, hair) or QIAGEN QIAamp Fast DNA Stool Mini kit (feces) (QIAGEN 2023). Extraction controls were included in each batch of extractions, replacing samples with double distilled water (ddH_2_O) to monitor for the detection of cross contamination. DNA from 390 samples was extracted within this study; this includes 262 collected samples in the GGASPA, 34 from the ex situ herd, 5 from known *C. bactrianus* , and 89 from previous sample collection (Silbermayr et al. 2010).

### 2.3 Analysis of Nuclear Genetic Diversity

Extracted DNA was used to amplify 16 autosomal polymorphic microsatellite markers (materials Appendix S1) previously shown to amplify in both *C. ferus* and *C. bactrianus* (Silbermayr et al. 2010); and four sex‐linked markers, three of which were previously validated (Felkel et al. 2019), whilst one was newly validated (Appendix S2). Of the four sex‐linked markers, three used polymorphisms for sexing and one was designed only to amplify in males. All 20 markers were genotyped using the multitube approach (Taberlet et al. 1996) to minimize error caused by allelic dropout (potentially more likely in lower‐quality samples obtained non‐invasively), with each PCR repeated three times and a negative control (ddH_2_0) included in each plate. PCR protocols are described in Appendix S3.

Genotypes were scored using GENEMAPPER v5.0 (Chatterji and Pachter 2006). Micro‐Checker was used to confirm absence of null alleles across the 16 loci (Van Oosterhout et al. 2004). Probability of identity (P(ID)) (Waits et al. 2001) was calculated using CERVUS 3.0.7 (Marshall et al. 1998).

### 2.4 Analysis of Mitochondrial DNA

Hybridization was tested on the maternal line using PCR‐RFLP on mitochondrial DNA (mtDNA) (Silbermayr et al. 2010). The Silbermayr et al. mitochondrial primer set amplifies an 804 bp fragment that contains a single nucleotide polymorphism (SNP) differing between *C. ferus* and *C. bactrianus* . A new primer set designed to amplify a shorter length (185 bp) fragment including the diagnostic SNP but more suitable for amplifying mtDNA from degraded fecal samples (Appendix S4). Amplification was assessed by gel electrophoresis and Sanger sequencing. The shorter mtDNA fragment containing the variable SNP was successfully amplified in 184 (93%) of the 198 samples used in this analysis (the full microsatellite data set of 257 samples included genotype data from 59 individuals from which it was not possible to obtain mtDNA for this study). Fourteen samples failed to amplify a clean sequence using the mtDNA marker.

### 2.5 Mitochondrial and Historic Introgression

Combining nuclear DNA and mitochondrial DNA genotypes helped to identify the source of introgression. If a microsatellite genotype profile suggested that a sample originated from a hybrid specimen, but the mtDNA sequence indicated *C. ferus* , then this implied the *C. bactrianus* genetic material was of paternal origin (paternal introgression). Conversely, if a microsatellite genotype profile suggested the individual to be a hybrid and the mtDNA sequence indicated *C. bactrianus* , it implied maternal introgression.

### 2.6 Population Genetic Summary Statistics

CERVUS 3.0.7 (Kalinowski et al. 2010; Marshall et al. 1998) was used to estimate the following measures of genetic diversity: number of alleles per locus; deviations from Hardy–Weinberg equilibrium; estimation of null allele frequencies; observed (H_O_) and expected (H_E_) heterozygosity. The presence of heterozygotes in known males and females of both species confirmed these markers as autosomal. After testing for deviation from normality (Shapiro–Wilk test), significant differences between H_O_ and H_E_ (Student *t*‐test and Hardy–Weinberg test) were tested in R (R Core Team 2026).

Bayesian clustering methods were used to determine shared ancestry proportions, identify potential *C. ferus* and *C. bactrianus* hybrids, and determine population structure (Pritchard et al. 2000). This method is appropriate as it uses multi‐locus genotypes (93% of the 16 loci were successfully amplified across all 257 samples) both to infer population structure and to assign individuals to populations using allele frequencies, without the need for prior classification. As samples were predominantly non‐invasive, it was important that classifications didn't require individual species visualization. STRUCTURE 2.3.4 (Pritchard et al. 2000) was run with correlated allele frequencies to detect distinct populations that are closely related for 1,000,000 iterations, with a burn‐in of 500,000 and 10 iterations, varying the number *K* of clusters between 2 and 10. STRUCTURE HARVESTER (Earl and vonHoldt 2012) and CLUMPAK (Kopelman et al. 2015) were then used to infer the optimal clustering solution (*K*) and to plot the results.

Levels of introgression of *C. bactrianus* DNA into *C. ferus* were assessed using a predetermined *K* = 2, with clusters representing each species. As known hybrids are present, admixture was assumed. Due to the close relatedness of the two species (Silbermayr et al. 2010) an allowance was made for up to 5% of ancestral alleles (qi > 0.95 for *C. ferus* , qi < 0.05 for *C. bactrianus* ) shared between the two species, considering individuals of either species with more than 5% (qi between 0.05 and 0.95) as hybrids. However, a threshold *q*‐value of 0.10 is often used amongst the conservation community to distinguish between pure and hybrid individuals after the publication of a methods comparison study by (Barilani et al. 2007; Smith et al. 2014; Vähä and Primmer 2006; Vanhaecke et al. 2012). Given that the genotype data does not include associated phenotype values for individuals, and in the absence of a standard threshold used by previous studies for camels, both threshold values are reported.

All pure *C. bactrianus* (qi < 0.05) and hybrid camels (qi between 0.90 and 0.10) (Vähä and Primmer 2006) were removed for the subsequent analysis of *C. ferus* population structure and summary statistics. The R package “Adegenet” (ADEGENET 2023) was used for principal component analysis (PCA) and to generate inbreeding coefficient (*F*_IS_) values.

Analysis of Molecular Variance (AMOVA) was used to partition proportions of genetic variation: (i) between populations (populations determined as: 1‐ pure *C. ferus* qi > 0.95, 2‐ pure *C. bactrianus* *qi* < *0.05*, 3‐ hybrids qi between 0.05 and 0.95 and 4‐ ex situ *C. ferus* ), (ii) between samples within populations, and (iii) within samples. AMOVA and FST were measured using GenAlEx (Peakall and Smouse 2006).

## 3 Ethics and Regulatory Approval

Ethics approval was obtained from the University of Kent's School of Anthropology and Conservation Research Ethics Committee (4‐PGR‐18/19). All samples were stored for international transportation in accordance with UK Animal and Plant Health Agency (APHA) regulations (dry with silica beads; APHA 2023), under APHA import permit ITIMP21.1842 and export permit No. 02231060279 from Customs General Admission of Mongolia.

## 4 Results

Of the total 449 samples available, 198 (45%) amplified successfully at a minimum of 75% of the microsatellite DNA loci and were therefore included in downstream analysis (5 blood, 105 fecal, 45 hair and 43 tissue, 34 of which were from ear tissue, 9 were from carcasses collected in the GGASPA). Of the 390 samples from which DNA was extracted during this study, 51% (*n* = 198) successfully amplified. This data set was combined with previous microsatellite genotyping data (*n* = 59) that used the same loci (Silbermayr et al. 2010) and included three samples in both datasets to harmonize allele lengths between laboratories. The final data set used for downstream analysis comprised 260 individuals genotyped at 16 microsatellite loci (“Dryad,” 2026). Three pairs of individuals were closely matched (P(ID) = < 0.0001), including one repeatedly collected reference sample, in which genotypes were 100% identical in the 14 loci that amplified (E6/Saran) used for quality control, indicating these pairs of samples were from the same individual. After removing one sample of the identified pairs (ID; Saran, AWC127, AWC135), respectively, the final data set comprised 257 individuals.

### 4.1 Nuclear Introgression

**FIGURE 2:**
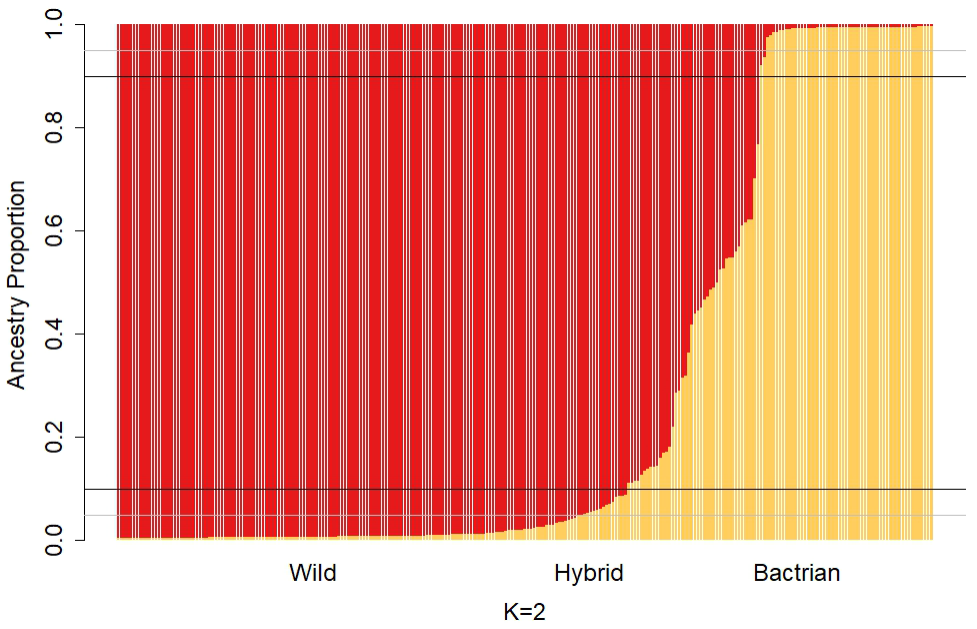
STRUCTURE analysis of *Camelus ferus* and *Camelus bactrianus* with pure/hybrid distinguishing thresholds. Each vertical line represents the ancestry proportion of one individual. Ancestry proportion is identified as *C. ferus* in red, with *C. bactrianus* in yellow. Black horizontal lines indicate qi = 0.10 and 0.90, grey horizontal lines identify qi = 0.05 and 0.95.5%, or less, of shared ancestral alleles (qi > 0.95 or qi < 0.05) or 10%, or less, shared ancestral alleles (qi > 0.90 or qi < 0.10) is considered “pure”. An individual with more than 5% (qi < 0.95) or 10% (qi < 0.90) is considered to be an introgressed or hybrid individual. These are the thresholds used for distinguishing between pure and hybrid species. Figure produced R using the *barplot* function (R Core Team 2026).

**FIGURE 3:**
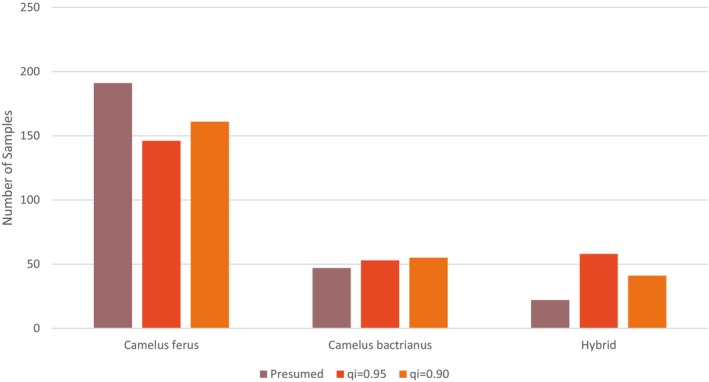
Total number of samples (*n* = 257) in the data set according to presumed species identification on collection of samples and genetically confirmed species identity after STRUCTURE analysis when applying a threshold of qi = 0.95 and qi = 0.90. From the 257 samples, species status presumed on collection were as follows: 47 samples from known *C. bactrianus* ; 188 samples from *C. ferus* (47 from the WCPF ex situ breeding center and 141 presumed *C. ferus* from the GGASPA); and 22 samples from known *C. bactrianus* × *C. ferus* hybrids, based on verified breeding history, collected from camel herders both in Mongolia and China. Results show that genotype STRUCTURE data can determine species and hybrids from non‐invasive samples and unidentified animals. This can be visualized in Figure 2.

Population structure with an assumed *K* = 2 was used to allow for classification of individuals of each of the two pure parental species and of the hybrid individuals along a continuum of introgression (Figure 2). Application of these thresholds for introgression determined the proportion of hybrids in the population. When a threshold of qi = 0.95 is applied across the full sample set (*n* = 257), 146 individuals are considered *C. ferus* (57%), 53 are *C. bactrianus* (20%), and 55 are hybrids (22%). When a threshold for introgression of qi = 0.90 is applied across the full data (*n* = 257), 161 (63%) are *C. ferus* , 55 (21%) are *C. bactrianus* , and 41 (16%) are hybrids (Figure 3).

To gain an initial understanding of introgression across the total *C. ferus* sample set, known hybrids were removed (samples of known hybrids collected from domestic herds; *n* = 22) and *C. bactrianus* (*n* = 47), leaving a total of 188 in situ and ex situ individuals. Of these 188 individuals, at qi = 0.95 19% (*n* = 58) are classified as hybrids, and at qi = 0.90 10% (*n* = 41) are classified as hybrids (Appendix S5).

Following this, samples determined pure *C. bactrianus* were removed from analysis. At a hybrid threshold qi = 0.95, 28% (*n* = 58) of wild samples are shown to be hybrids, whilst at a hybrid threshold of qi = 0.90, 20% (*n* = 41) of the *C. ferus* samples are hybrids (Table 1).

**TABLE 1:** Population statistics for the different camel populations.

Population	*N*	Mean Hobs	Mean Hexp	HW mean *p*	Mean difference (95% CI)	*t* test *p*	FIS	Mean *F* (TrioML) Comb
**qi = 0.95**								
Full data set	260	0.45	0.57	0.0013	0.12 (0.08‐Inf)	< 0.0001	0.21 (0.13–0.27)	0.23
*C. ferus*	106	0.39	0.42	0.42	−0.03 (−0.07‐Inf)	0.05	0.09 (0.01–0.18)	0.25
*C. bactrianus*	53	0.5	0.56	0.26	0.07 (−0.006‐ Inf)	0.06	0.13 (0.03–0.24)	0.29
Ex situ	48	0.39	0.37	0.64	−0.02 (−0.04‐ Inf)	0.92	−0.04 (−0.09–0.03)	0.2
Hybrids	58	0.57	0.6	0.39	0.03 (−0.01‐ Inf)	0.1	0.06 (< −0.01–0.13)	0.14
**qi = 0.90**								
Full data set	257	0.46	0.58	0.001	0.12 (0.08‐ Inf)	< 0.0001	0.21 (0.13–0.27)	0.23
*C. ferus*	116	0.4	0.44	0.38	0.04 (0.004‐Inf)	0.03	0.07 (0.01–0.15)	0.24
*C. bactrianus*	55	0.5	0.56	0.18	0.07 (−0.005‐Inf)	0.06	0.13 (0.01–0.24)	0.29
Ex situ	47	0.39	0.37	0.65	−0.02 (−0.04‐Inf)	0.91	−0.04 (−0.1–0.04)	0.21
Hybrids	41	0.62	0.61	0.46	−0.004 (−0.05‐ Inf)	0.56	0.0075 (−0.1–0.08)	0.1

*Note:* Populations were determined using both STRUCTURE data and sample collection data and then further determined and analysed for the two different hybrid thresholds qi = 095 and qi = 0.90. For each population the following population statistics were generated: Mean Hobs and Mean Hexp = Mean observed and expected heterozygosities; HW mean *p* value = for which the null hypothesis is that the population is not deviating from the Hardy Weinberg equilibrium; Mean difference (95% CI): Paired *t*‐test (one‐tailed, alpha = 0.05) *t*‐test *p*‐value = testing for significant differences between H_O_ and H_E_, FIS = the inbreeding coefficient including 95% confidence intervals is the deviation of Hobs of an individual relative to the expected heterozygosity under random mating‐FIS > 0 more inbreeding than expected, FIS = 0 No inbreeding, FIS < 0 Less inbreeding than expected. Mean *F*, the inbreeding co‐efficient as the probability of 2 homologous genes in an individual being identical by descent. *F* gained using Coancestry (TrioML).

**FIGURE 4:**
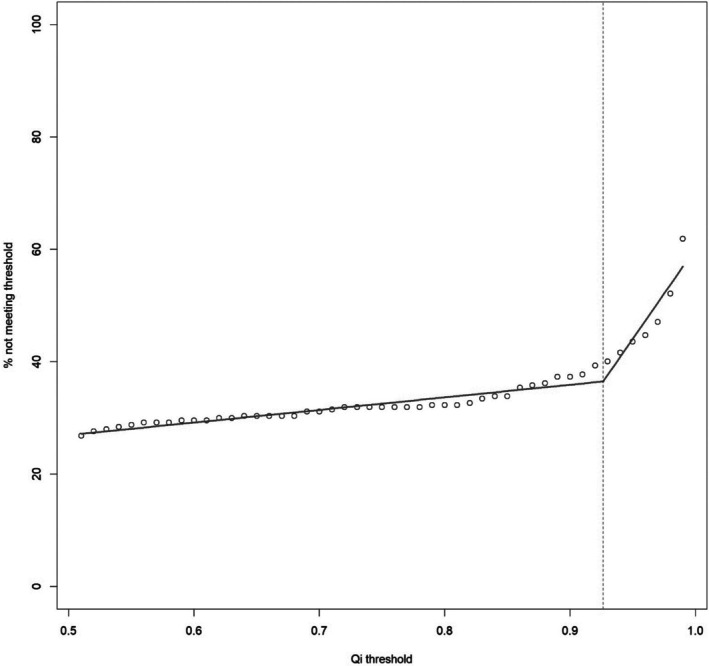
Proportion of population considered hybrids as a function of Qi threshold for “pure” species. Data points represent empirical proportions of the sample considered hybrids at different threshold Qi values for hybrid status. This will, by definition, be a monotonically increasing function. A data‐driven estimate of threshold for hybrid status is provided using segmented linear regression, assuming that below this value the proportion of the sampled population deemed hybrids will be relatively robust to changes in threshold, whereas above this value the numbers classified as hybrids will vary widely with small changes in (arbitrary) threshold. It is therefore estimated that this value as Qi = 0.9264, using segmented linear regression assuming a single break point. The solid line presents the estimated piecewise regression curves, and the dashed line the estimated break point for change.

To visualize the variation in hybrid proportion when applying different qi thresholds, qi hybrid threshold was plotted against the proportion of individuals in the full dataset deemed hybrid at that threshold. From visual inspection, this shows a transition point at around a hybrid threshold of qi = 0.93; this was supported by a data‐driven estimate of the threshold using segmented linear regression (Muggeo 2003) with an assumed single break point which provided a regression break point estimate of 0.926. This supports both the conventional threshold previously used in the literature and the conservative introgression estimate (Figure 4).

**FIGURE 5:**
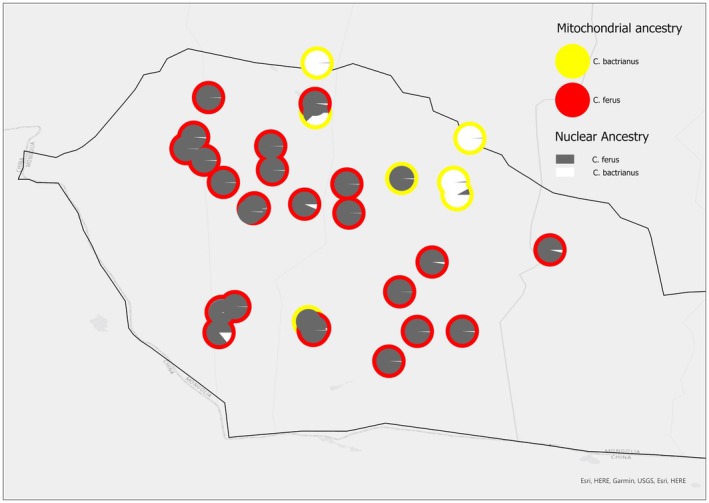
STRUCTURE results (*K* = 2) for C. ferus and C. bactrianus shown in collection location in GGASPA. Each pie chart represents an individual sample, plotting the proportions of ancestry from *C. ferus* alleles in red, with the proportion of *C. bactrianus* alleles in yellow. As pie charts are plotted at the location where the samples were collected, it allows for monitoring of introgression geographically across the GGASPA.

To gain a representation of the in situ population of the *C. ferus* , only those samples collected in the GGASPA were analysed. Of these *n* = 103 individuals, 18% (*n* = 19) could be considered hybrids when a qi = 0.95 threshold is applied. With a threshold of qi = 0.90 applied, 10% (*n* = 10) could be considered a hybrid. This introgression is distributed across the GGASPA (Figure 5). The current GGASPA population size estimate is 664, 95% CI = 440–1100 (Jemmett 2024). Of the 103 samples collected in the GGASPA and successfully amplified, 97 are from pure or hybrid *C. ferus* . Therefore, this genotyped dataset could represent up to 15% of the in situ population.

Of the 47 ex situ samples used in this analysis, two sampled individuals (of which one is a current herd member) show introgression at qi = 0.90 and five (of which three are current herd members) show introgression at qi = 0.95.

### 4.2 Mitochondrial and Historic Introgression

A total of 41 hybrids were initially identified using the microsatellite data (qi = 0.90), 36 amplified successfully using the mitochondrial assay (Appendices S5 and 10). The mtDNA results indicated that 17 were maternally inherited and 19 paternally inherited. Sex information, obtained using the sex‐linked markers (Appendix S6), identified that of the 17 hybrids that showed maternally inherited introgression, 10 were female (59%) and could therefore further pass on *C. bactrianus* mtDNA. Of the 58 hybrids determined using the qi threshold of qi = 0.95, 52 (90%) amplified successfully using mtDNA. These results suggested that 32 (62%) are paternally inherited hybrids, whereas 20 (38%) are maternally inherited; of these, 11 (21%) are female and so can pass on *C. bactrianus* mtDNA.

**FIGURE 6:**
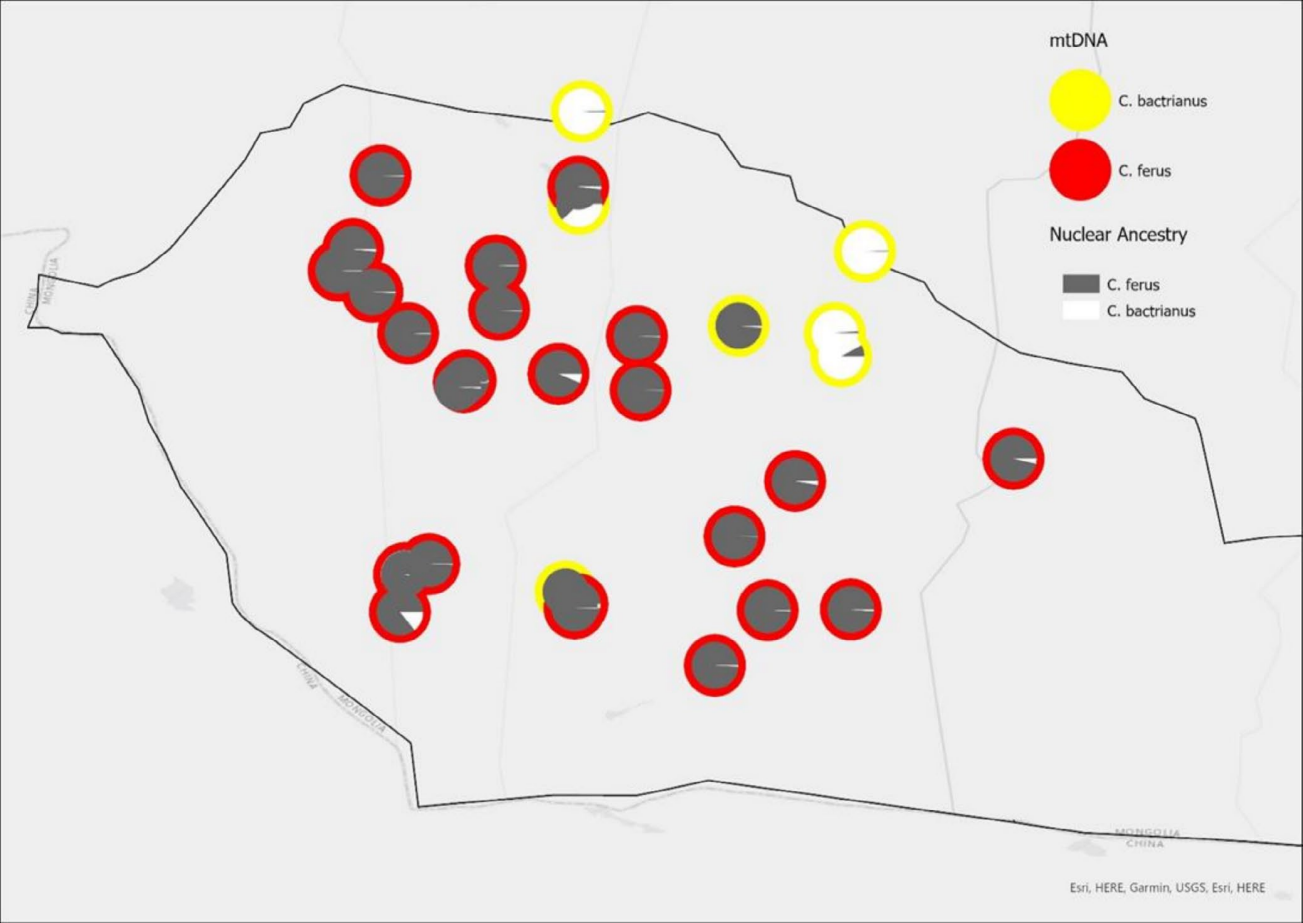
Mitochondrial introgression across the GGASPA. Each pie chart represents an individual, showing the nuclear DNA‐derived proportions of ancestry from *C. ferus* alleles in grey and the proportion of *C. bactrianus* alleles in white. The outline color of each pie chart represents mtDNA evidence for maternal introgression—Yellow for *C. bactrianus* and red for *C. ferus* . Pie charts are plotted at the location where the individuals were sampled.

Eight samples initially considered “pure” *C. ferus* based on the nuclear DNA results (qi > 0.90) and on the basis of their being collected from within the GGASPA show introgression in their mtDNA (Figure 6, Appendix S10). The extent of nuclear introgression within these mtDNA‐typed individuals ranges from 0.947 to 0.994.

### 4.3 Heterozygosity and Inbreeding

Across the 16 autosomal microsatellite loci, H_O_ ranged from 0.03 to 0.85 (mean = 0.39, standard deviation = 0.21; Appendix S1). Null alleles were not present in any of the 16 loci. The estimated null allele frequency (*F*) ranged from −0.0016 to 0.179 (Appendix S7). Population summary statistics were calculated separately for the ‘pure’ *C. ferus* individuals sampled in the Great Gobi Strictly Protected Area A (*n* = 106–116, depending on hybrid threshold, Table 1) after removing the data derived from individuals obtained from the WCPF breeding center and the detectable hybrids (qi = 0.90 was used here as it incorporated hybrids at both thresholds). This partitioning of the data ensured that analysis of genetic composition was not distorted by either mixing data from individuals of two different species or by inadvertently including data from closely related ex situ animals, thereby enabling more appropriate comparisons of genetic diversity in *C. ferus*. Alleles per locus ranged from 3 to 16 (Appendix S9).

Analyses of genetic diversity revealed that the only population to deviate significantly from HWE was the full data set, a result that was not unexpected given that it contains both parent species and hybrids. All other populations did not deviate significantly from HWE (Table 1). However, when a threshold of 10% shared ancestry was applied, this resulted in a significant difference between the H_O_ and H_E_ in the wild population (*t‐*test *p* value: 0.03). This result suggests that although in HWE, *C. ferus* is showing a difference in observed heterozygosity that is not due to chance alone and is showing lower levels of heterozygosity than expected. It may also suggest that a 10% threshold does indeed indicate introgression. The former conclusion is supported by *F*_is_ values, that suggest more inbreeding than expected at random in all populations other than the ex situ population. The latter is also supported as there is an increased observed heterozygosity in the 0.90 data set (0.4) compared to the 0.95 data set (0.39) (Table 1) which may be due to the recent introduction of alleles that have not yet reached Hardy Weinberg equilibrium. When the percentage of the full data set (*n* = 257) that did not meet a given threshold was plotted against qi values between 50% and 100% shared ancestry, segmented linear regression suggested the breakpoint was qi = 0.92 (Figure 4). Therefore, at a 10% threshold some introgression could be expected.

Allelic richness was significantly different between the populations (Kruskal‐Wallis chi‐squared = 20.097, df = 2, *p*‐value = < 0.0001). Allelic richness is greater in the wild population (median 10) compared to the ex situ population (median = 6), (Unpaired Mann Whitney; *C. ferus* /Ex situ *p*‐value = 0.0005378, *C. ferus* /Hybrid *p*‐value = 0.479, Ex situ/hybrid *p*‐value = < 0.0001), a result likely due to both the larger‐sized wild population that is spread over a vast geographic area in comparison to the ex situ herd which originates from a small number of related founders, and is therefore likely to have been impacted by effects of random genetic drift.

**FIGURE 7:**
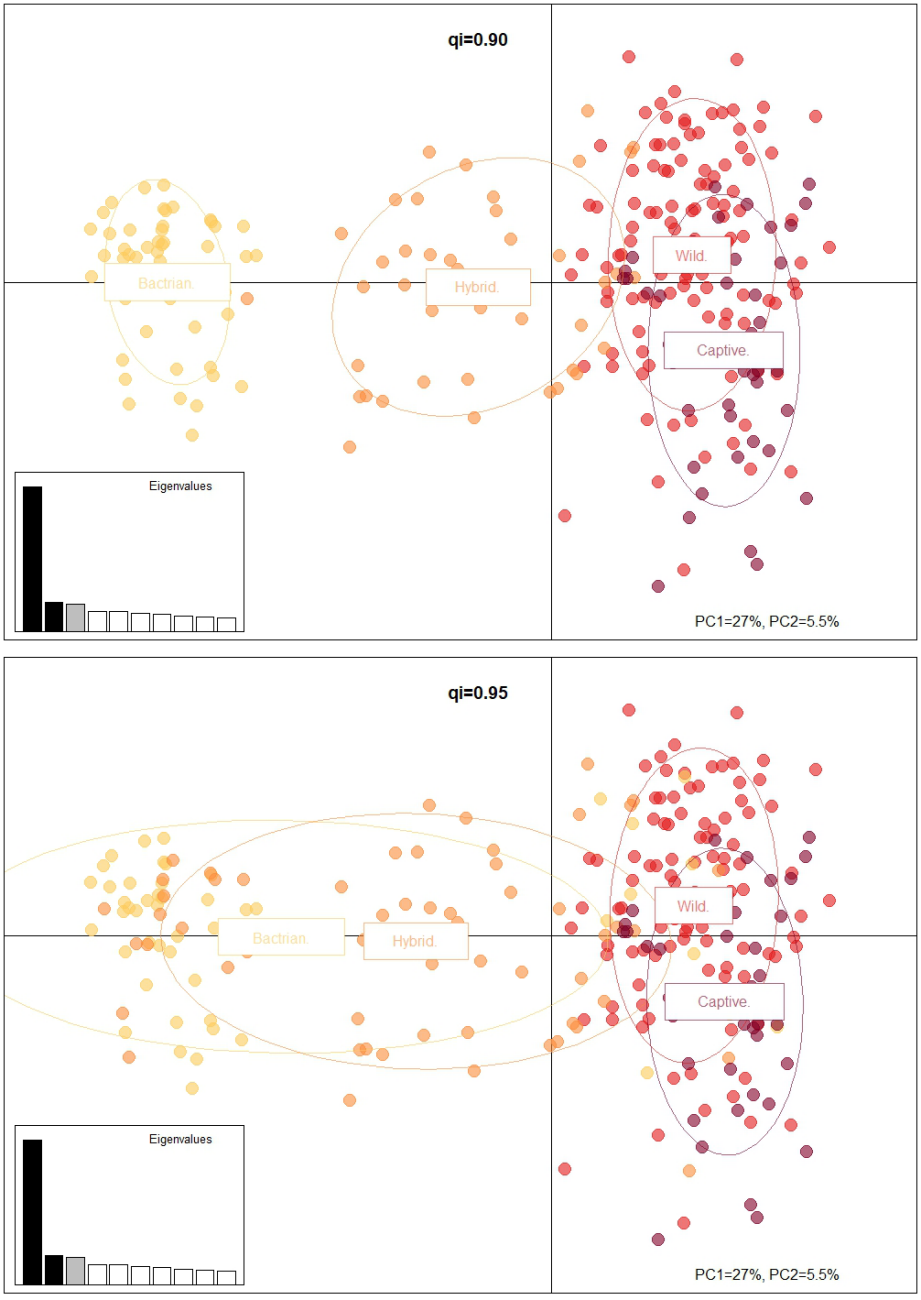
Principal component analysis (PCA) including all 257 samples. Discrete color relates to populations: *C. ferus* , *C bactrianus*, hybrids and ex situ. Populations determined after STRUCTURE analysis using a qi value of 0.90 (top) and qi = 0.95 (bottom) to determine hybrids. A qi = 090 suggests more discrete populations whereas a qi = 0.95 shows an increased overlap. Figure produced with Adegenet. PC1 = 27% and PC2 = 5.5%.

Results also suggest there is no significant difference between the inbreeding coefficients (*F*, TrioML) for the ex situ herd when compared to the in situ (Unpaired Mann Whitney: Wild/Ex situ *p* value = 0.5158). There is however a significant difference between the inbreeding coefficients of all other groups (*F*, TrioML Unpaired Mann Whitney: Wild/Hybrid *p*‐value = < 0.0001, Ex situ/hybrid *p*‐value = 0.0007) which suggests that despite allelic richness being greater in the wild compared to ex situ, the genetic composition and extent of inbreeding observed in the wild population is no different to that in the ex situ population (Figure 7).

Analysis of molecular variance AMOVA showed that the greatest variance was within individuals (75%), then among populations (21%) and finally among individuals (1%) (Appendix S8). This partitioning of variance in genetic diversity reflects the overlap between all these populations. The largest *F*_ST_ values are between the *C. bactrianus* and the ex situ population (*F*_ST_ = 0.397), with *C. ferus* and *C. bactrianus* showing similar values (*F*_ST_ = 0.372). Hybrids show higher *F*_ST_ when compared to *C. bactrianus* (*F*_ST_ = 0.165) than *C. ferus* (*F*_ST_ = 0.074) or ex situ (*F*_ST_ = 0.105).

## 5 Discussion

Non‐invasive sampling combined with genetic monitoring has enabled a greater understanding of the extent and sources of introgression in *Camelus ferus* in Mongolia and how this contributes to observed levels of genetic diversity. This genetic perspective is crucial to understanding the hybridization problem and is an important first step toward identifying options for conservation management. Evidence of both nuclear, mitochondrial, and historic introgression of *C. bactrianus* genes in the *C. ferus* population is shown across the GGASPA and in some individuals within the ex situ herd, the true extent of which is determined by which threshold of introgression is applied. The results also reveal reduced heterozygosity and increased inbreeding in the in situ population of *C. ferus* in the GGASPA and show that these levels of lower heterozygosity are present in the ex situ herd.

### 5.1 Managing Introgression in Ex Situ Populations

Ex situ populations are often managed with the aim of preserving taxonomic integrity and providing a potential future source of individuals for reintroduction, so understanding the true conservation value of these populations is important. This is true for *C. ferus*; prior to this study, genetic diversity, relatedness and hybridization values for the ex situ herd of *C. ferus* were unknown. Results show that allelic richness is greater in the wild than in the ex situ population, a result likely due to a larger‐sized wild population that is spread over a vast geographic area in comparison to the ex situ herd which has come from a small number of founders and likely suffers high levels of genetic drift. The higher variance between the *C. bactrianus* and the ex situ individuals than *C. bactrianus* and in situ *C. ferus* (AMOVA, Appendix S8) could be due to the effect of recent genetic drift on the exsitu population, with allelic differences being more pronounced in the ex situ population when compared to the in situ population. This is supported by the allelic richness and inbreeding results previously reported. Despite this, the results show that the genetic composition and extent of inbreeding observed in the wild population is no different to that in the ex situ population.

The analysis also identified three individuals showing introgression in the ex situ herd. As none of the ex situ herd show evidence of introgression from their maternal mitochondrial DNA, it is concluded that this introgression has come from the paternal line (i.e., a bull *C. bactrianus* breeding with a female ex situ *C. ferus* ). The *C. ferus* ex situ population is closed; breeding is determined by animals being added to or removed from the population. This procedure allows for breeding management that maintains diversity and where hybrids can be easily isolated from breeding. As this ex situ population is considered an insurance population for this endangered species, monitoring hybridization and genetic diversity in the ex situ herd could help to evaluate population integrity and inform species recovery.

### 5.2 Managing Introgression in In Situ Populations

Introgression in threatened species from domestic species is usually due to range overlap (Iacolina et al. 2019). Climate change is causing increased desertification and drought in the Gobi, and this, combined with overgrazing and an increase in human activities such as mining (Han et al. 2021), is increasing competition for resources and therefore promoting range overlap between *C. ferus* and *C. bactrianus* . Local nomadic herding practices also increase the frequency of contact, and therefore the probability of mating between the two species is more likely. Exacerbating this problem is the small population size of *C. ferus* that restricts mate choice, and the compatible breeding behaviors between the two species. For these reasons in situ hybridization was anticipated in the in situ *C. ferus* population. In Mongolia *C. ferus* is range‐restricted to the GGASPA, a 45,000 km^2^ closed National Park, established to protect this species. Despite the vast size of this un‐fenced park, and the estimated 600 individuals that live in it (Jemmett 2024), this study confirms that introgression can be seen throughout (Figures 5 and 6).

### 5.3 Drivers of Introgression

Mitochondrial data gained in this study allowed us to observe hybridization patterns and the potential drivers producing them. Of the paternally inherited hybrids (*n* = 19), the majority (79%) were from “wild” samples collected from either the GGASPA or China. This finding suggests bull *C. bactrianus* camels are entering the GGASPA and mating with female *C. ferus* . The *F*_ST_ values support this interpretation, with results showing that hybrids are closer in variance to the in situ population than to *C. bactrianus* . Conversely, the majority (82%) of the maternal hybrids are from domestic *C. bactrianus* herds in China, suggesting a *C. ferus* bull mating with herded *C. bactrianus* females. Camels are a rutting species, with one bull holding and fighting for a number of females, so these results make both ecological and circumstantial sense: the introgression that was detected could be originating from bulls traveling to find females: *C. bactrianus* bulls dispersing into the national park and *C. ferus* bulls dispersing out of the park. This scenario is very likely exacerbated by a number of factors: the GGASPA is unfenced (and unlikely to be fenced due to its scale), local practices of nomadic herding methods, compatible mating behaviors and increased competition for resources (Silbermayr et al. 2010).

### 5.4 Hybrid Thresholds

Defining an individual as a “hybrid” or “pure” based on its proportion of introgression or admixture is not straightforward. The proportion of admixture present in a population considered to represent ‘purity’ is ultimately arbitrary, set by the perspective and interests of those managing the population; furthermore, the accuracy of admixture measurement will depend on the quality of DNA extracted, the sensitivity and diagnostic ability of markers used, and the availability of other species information.

Previous genetic analysis can help determine threshold (Smith et al. 2014) as can phenotype data (Schrey et al. 2007). With *C. ferus* there was neither previous genetic data to work with nor phenotypic data. Therefore, it was decided to set both a 10% (qi = 0.90) and 5% (qi = 0.95) threshold. The 10% threshold was included as this is a frequently cited modeled threshold (Vähä and Primmer 2006) that is widely used in other studies (Barilani et al. 2007; Vanhaecke et al. 2012). The 5% threshold, although a lower estimate, is one that was considered more realistic. When segmented linear regression was used, a single break point of qi = 0.9264 was suggested—between these two thresholds. Although these differing thresholds generate different extents of hybridization, it is clear introgression from *C. bactrianus* can be seen across the Mongolian in situ population, ranging from 10% to 22% of the wild population.

Further complicating the definition of “pure” when describing an individual's provenance is the possibility of historic introgression. It has been suggested that interspecific hybridization has occurred throughout the evolutionary history of the *Camelus* genus, with hybridization widespread between *C. ferus* , *C. bactrianus* and the extinct *C. knoblochi* (Yuan et al. 2014). Historic introgression was inferred in *C. ferus* using mitochondrial DNA. When comparing nuclear results to mitochondrial, eight of the samples show no introgression at a nuclear level, but do show introgression in mtDNA, suggesting that this introgression could be historic. This finding has been observed in other species (Allendorf et al. 2001) including other camelids (Almathen et al. 2016) and previously in *C. ferus* itself where the mtDNA haplotype was wild but the Y haplotype was *C. bactrianus* (Felkel et al. 2019). Given that mtDNA provides a longer‐term picture of evolutionary ancestry, where introgression is observed in the mtDNA genome of individuals deemed to be ‘pure’ from nuclear DNA, they are likely to be the product of historical hybridization. This may also further exemplify the arbitrary nature of the nuclear thresholds used.

### 5.5 Managing Hybridization

Managing hybridization is an ongoing challenge. As climate change, habitat loss and degradation increase, the opportunity for anthropogenic hybridization also increases. Whilst in species for which outbreeding depression threatens their genetic integrity and adaptive potential, limiting introgression can be especially important, it is also true that in some small or isolated threatened populations suffering from low genetic diversity and inbreeding depression, successful restoration of the original population genetic variation may not be possible using traditional population recovery methods. In these cases, genetic rescue of populations may be required (Edelman and Mallet 2021).

While extinction is forever, preserving hybrids could be advantageous, whether that be as a means of preventing overall extinction (Allendorf et al. 2001; Der Sarkissian et al. 2015; Gese et al. 2015), acting as a reservoir to preserve the parental species' genetic material (Chan et al. 2019; Lawson et al. 2023), fulfilling important ecological functions (Steiner et al. 2017) or leading to adaptive traits which may allow for expansion into new niches and ranges (Chan et al. 2019)—an ability that will become increasingly important as environments change.

Discussion around the conservation of hybrids remains controversial, but if a species, showing introgression or admixture, holds the role of fulfilling an ecological function, is it not an evolutionary entity, worthy of protection? Especially when the thresholds used to determine these as “hybrids” may be arbitrary. Previous research suggested that genetic diversity is reduced in the population of the Wild Camel (Lado et al. 2020) and results from this study confirm this to be true in the GGASPA population. Introgression could either improve or impair this diversity. As more is learnt about hybridization, species thresholds and environmental threats, the ability to make informed decisions will be improved.

In the case of *C. ferus* , our understanding of the extent of introgression across the GGASPA has increased, both in nuclear and mitochondrial genomes. The knowledge that between 10% and 22% of the in situ population show introgression may be cause for concern. But as interspecific hybridization has been shown to occur throughout the evolutionary history of the *Camelus genus*, further work involving whole genome sequencing is required to fully understand the extent of any impact or opportunities.

This study has also shown that there are no geographic barriers preventing this introgression and that the source is likely from *C. bactrianus* bulls entering the GGASPA. Although deciding what to do about existing cryptic hybrids in a vast unfenced desert is difficult, there may be an opportunity to limit further introgression by working with local herdsmen, the GGASPA ranger service and Mongolian Authorities to limit the number of these *C. bactrianus* bulls entering the park. Further to this additional study and education programmes are required in the buffer zone of the GGASPA to determine local perception and opinion on *C. bactrianus* × *C. ferus* hybridization.

*C. ferus* remains endangered; whilst it is not fully understood whether hybridization poses a threat or an opportunity for conserving this species, in situ, managers will now be better informed to make key decisions.

## Author Contributions

**Anna M. Jemmett:** conceptualisation (equal), field work (primary), lab work (primary), data analysis (primary) and writing (primary). **John G. Ewen:** conceptualisation (equal), field work (support). **Adiya Yadamsuren:** conceptualisation (support), field work (support). **Deborah A. Dawson:** lab work (advisory support) and data analysis (equal). **Lucy Raggett:** mitochondrial sequencing analysis (equal). **Pamela A. Burger:** conceptualisation (equal), lab work (advisory support) and data analysis (equal). **Jim. J. Groombridge:** conceptualisation (equal), lab work (advisory support) and data analysis (equal).

## Funding

This research was funded by the Wild Camel Protection Foundation (WCPF) with the laboratory work supported by both the University of Veterinary Medicine Vienna, the Austrian Science Fund FWF (P29623‐B25) and the UK Natural Environment Research Council (NERC) Environmental Omics Facility (NEOF1257).

## Disclosure

Benefit‐Sharing Statement: A research collaboration was developed with scientists from Mongolia, including representatives from the Government of Mongolia. These in situ partners provided genetic samples and logistical support for the first author to collect additional samples. All collaborators are included as co‐authors; the results of this research have been shared with the provider communities and the broader scientific community, and the research addresses a priority concern, in this case the conservation of the Wild Camel. More broadly, our group is committed to international scientific partnerships, as well as institutional capacity building.

## Conflicts of Interest

The authors declare no conflicts of interest.

## Supporting information


**Appendix S1:** ece373293‐sup‐0001‐AppendixS1.xlsx.

**Appendix S2:** ece373293‐sup‐0002‐AppendixS2.xlsx.

**Appendix S3:** ece373293‐sup‐0003‐AppendicesS3‐S4.docx.
**Appendix S4:** ece373293‐sup‐0003‐AppendicesS3‐S4.docx.

**Appendix S5:** ece373293‐sup‐0004‐AppendixS5.xlsx.

**Appendix S6:** ece373293‐sup‐0005‐AppendixS6.docx.

**Appendix S7:** ece373293‐sup‐0006‐AppendicesS7‐S10.docx.
**Appendix S8:** ece373293‐sup‐0006‐AppendicesS7‐S10.docx.
**Appendix S9:** ece373293‐sup‐0006‐AppendicesS7‐S10.docx.
**Appendix S10:** ece373293‐sup‐0006‐AppendicesS7‐S10.docx.

**Appendix S11:** ece373293‐sup‐0007‐AppendixS11.docx.

## Data Availability

Genetic data: Individual genotype data are available on Dryad: https://doi.org/10.5061/dryad.d51c5b0j9. The mtDNA alignment is available in Appendix S4 and the sex‐linked marker alignments in Appendix S2.
