# Supplementary material for: Challenges of Introgression in Conservation: Genetic Diversity of the Endangered Wild Camel (Camelus ferus) in Mongolia
Source: Ecol Evol. 2026 Mar 29;16(4):e73293. doi: 10.1002/ece3.73293 (PMC13107281; doi:10.1002/ece3.73293)
Supplement: Supplementary file 3 — Appendix S3: ece373293‐sup‐0003‐AppendicesS3‐S4.docx. Appendix S4: ece373293‐sup‐0003‐AppendicesS3‐S4.docx. [file ECE3-16-e73293-s006.docx]

**Annex 3- PCR Protocol**

**Msat:** All 20 markers used the multitube approach to minimize error caused by allelic dropout, with each PCR repeated 3 times and a negative control (ddH20) included in each plate. 3 μl volume PCRs used the following reagents: 1 μl DNA, 1 μl of primer mix (23 μl Low TE (10mM Tris-HCl (pH 8.0) and 0.1mM EDTA (pH 8.0)), 1 μl forward and 1 μl reverse primers (both at 5 uM) and 1 μl QIAGEN Multiplex PCR Master Mix (supplied with the QIAGEN Multiplex PCR Kit, Cat. No. / ID: 206145). PCR protocols depend on multiplex/annealing temperature (see supplementary material 1)

**QMix56**: Incubate at 95 degrees for 15 min, 45 cycles of 94 degrees for 30 secs, 56 degrees for 90 sec, 72 degrees for 60 sec; Incubate at 60 degrees for 30 min.

**QMix60**: Incubate at 95 degrees for 15 min, 35 cycles of 94 degrees for 30 seconds, 60 degrees for 30 seconds, 72 degrees for 45 seconds. Incubate at 72 degrees for 10 min.

**MtDNA:**

Reactions used the following reagents: 3μL DNA, 0.4μL forward primer, 0.4μL Reverse primer, 10μL Red Taq, 2.2μL double distilled water and 4μL diluted Bovine Serum Albumin (BSA) (1μL BSA - Acetylated; 99μL double distilled water). Negative controls, where DNA was replaced with ddH_2_O, were included in each plate. After a PCR protocol of 92°C for 8 min, 45 cycles of: 95°C for 30 seconds, 52°C for 45 seconds, 72°C for 45 seconds and a final extension of 72°C for 10 min, PCR product was digested with: 1 unit of *Xmil*, 10x Buffer B (with BSA) and double distilled water at 37°C for 1hr

**Annex 4: Analysis of MtDNA**

We used the “HYB 185 bp” primer pair (forward 5’- GTT CAT CGT AAT CGG CCA AGC -3’ and reverse 5’- GGC CTC TTC CCT GAG TCT TAG -3’) in 20μL volume PCRs to amplify a 185bp mtDNA fragment containing the single nucleotide polymorphism that is diagnostic for either *C. ferus* or *C. bactrianus* (supplentary information for PCR protocol). This digestion was either analysed by gel electrophoresis or the DNA fragment was sequenced following the Macrogen EZ-Seq protocol for purified PCR product of less than 300bp (Macrogen, Inc.). For gel electrophoresis, the presence of two bands at 60bp and 120bp indicated *C. bactrianus* maternal lineage, whereas an uncut single band of 185bp indicated a *C. ferus* maternal lineage. Using DNA sequence to verify maternal lineage, the base motifs differ between species, with “CATATGAT” for *C. bactrianus* and “TATATGAT” for *C. ferus.*
