# Supplementary material for: Challenges of Introgression in Conservation: Genetic Diversity of the Endangered Wild Camel (Camelus ferus) in Mongolia
Source: Ecol Evol. 2026 Mar 29;16(4):e73293. doi: 10.1002/ece3.73293 (PMC13107281; doi:10.1002/ece3.73293)
Supplement: Supplementary file 5 — Appendix S6: ece373293‐sup‐0005‐AppendixS6.docx. [file ECE3-16-e73293-s001.docx]

**Annex 6: Sex linked marker development, validation and sexing results**

- 3 Y linked primers (COO, CJT, CJE) were used for sexing camel species (*Camelus ferus* and *Camelus bactrianus*). Three primer sets used were taken from Felkel et al 2019 [21], but individuals were genotyped with fluorescently labelled primers on an ABI3730 DNA Analyzer rather than running on an agarose gel as was done by Felkel et al 2019. The use of QIAGEN multiplex master mix also enable additional previously un detected homologs to be amplified on both sex chromosomes increasing the accuracy and utility of these primer sets for sexing (Table 1)
- ABI genotypinging revealed that 2 markers (CJT and CJE) amplify in males and females indicating they have homologs on the X and Y chromosomes.
- 1 new sexmarker (C11) was designed from an X-linked contig (NCBI: BCGSAC_Cfer_1.0) isolated from the camel genome and was found to only amplify the X chromosome but was variable so able to identify some females (those that were heterozygous) allowing individuals to be sexed when combined with other Y linked markers. C11 does not amplify a Y chromosome homolog (Table 1)
- Used initially for testing on known sex individuals- captive animals N=34 (19 Female, 15 male). Of these 85% agree with sex based on morphology, none disagreed.

Then used across full available sample set (N=163).

Table 5- Sex Marker information

| **Sex Marker** | **Fluro-dye** | **Primer Sequences** | **Allele sizes of the X and Y chr homologs (bp)** | **Sequence from which marker was designed (GenBank sequence accession number /Reference)** |
| --- | --- | --- | --- | --- |
| COO | Hex | F: TAGTCTGCAGCTCCTGGTCA | Y=170;  No amplification in X chr | CBacY1775_contig157:1502 (170bp) |
|  |  | R:ATTTGCCAGGCTAACAATGG |  |  |
| CJT | Hex | F:ATATCCCAGGCACTGCTGAA | X = 146, y=144 | CBacY1775_contig185:3779 (146bp) |
|  |  | R: ATTAGCGGATTTCCCTCTGC |  |  |
| CJE | 6Fam | F:GTCTTGGTCAGGGATTGCAT | X=181; Y=178 | CBacY1775_contig57:6691 (178 bp) |
|  |  | R: CTCTTAGCCCTTGCATCTGG |  |  |
| C11 | 6Fam | F: CACAGACATGTGTGCCATC | No amplificaton in Y chr; In X chr. ranges from 198 – 222 | NCBI: BCGSAC_Cfer_1.0 |
|  |  | R:AAAGCAAATGGAAGATGCTC |  |  |

**Known sex samples**

Methods

34 tissue samples collected from known sex individuals a captive herd of wild camels (*Camelus ferus*) in Mongolia. Tissue samples collected by a registered veterinarian using Dalton flexo-DNA ear tags during standard veterinary ear tagging procedures. DNA extracted using Qiagen DNeasy blood and tissue kits. Extracted DNA was used to amplify the 4 sex linked markers using the multitube approach, with each PCR repeated 3 times and a negative control (ddH20) included in each plate. 3ul volume PCRs were performed with the following:: 1 μl DNA, 1 μl of primer mix (23 μl Low TE ( 10mM Tris-HCl (pH 8.0) and 0.1mM EDTA (pH 8.0).), 1 μl forward and 1 μl reverse primers (both at 0.2? uM) and 1 μl QIAGEN Multiplex PCR Master Mix (supplied with the QIAGEN Multiplex PCR Kit, Cat. No. / ID: 206145). The PCR was incubated at 95 degrees for 15 min, 35 cycles of 95 degrees for 30 seconds, 60 degrees for 30 seconds, 72 degrees for 40 seconds. Incubate at 72 degrees for 4 mins. Genotypes were scored using the Genemapper software. Each sample was tested with each marker 3 times. For that sample to be scored it must show at least 2 matching repeats and no mismatches.

COO- a 170 bp amplicon indicates the presence of a Y chromosome, indicating the individual is male (the X chromosome does not amplify)

CTJ- shows a 146 bp amplicon in the presence of an X chromosome, indicating the individual is female and shows a 144 bp amplicon in the presence of an Y chromosome, indicating the individual is male.

CJE- shows a 181 bp amplicon in the presence of an X chromosome, indicating the individual is female and shows a 178 bp amplicon in the presence of an Y chromosome, indicating the individual is male.

C11- is X-linked (Y chromosome doesn’t not amplify) and so considered female (XX) if heterozygote. Males (XY) are always homozygotes and cannot be heterozygotes since males have only one X chromosome. Females can also be homozygotes, but any heterozygotes must be female for marker C11. Homozygotes were not sexed, since homozygotes could be female or male for this marker. All heterozygotes were scored as female.

Results

Table 6: Results from known sex samples (N=34). All samples from the ex-situ herd of Wild Camel. Samples are tissue, collected during routine veterinary eartagging.

| ID | Known sex (based on morphology) | COO_  Y=170  X-no amp. | CTJ_  X=146  Y=144 | CJE_  X=181  Y=178 | C11_  X-linked  Y=no amp. | Sex based on sex markers combined | How many markers agree with sex based on morphology* | How many markers disagree |
| --- | --- | --- | --- | --- | --- | --- | --- | --- |
| E1 | Male | Y | Y | Y | Homo | Male | 3 | 0 |
| E2 | Female |  |  | X |  | Female | 1 | 0 |
| E3 | Female |  | X | X | Het=Female | Female | 3 | 0 |
| E4 | Female |  | X | X | Het=Female | Female | 3 | 0 |
| E5 | Male | Y | Y |  | Homo | Male | 2 | 0 |
| E6 | Female |  | X | X | Het=Female | Female | 3 | 0 |
| E7 | Male | Y | Y | Y | Homo | Male | 3 | 0 |
| E8 | Female |  | X | X | Homo | Female | 2 | 0 |
| E9 | Male |  |  |  | Homo | Unknown |  |  |
| E10 | Female |  | X | X | Het=Female | Female | 3 | 0 |
| E11 | Female |  | X | X | Het=Female | Female | 3 | 0 |
| E12 | Male | Y | Y | Y | Homo | Male | 3 | 0 |
| E13 | Female |  | X | X |  | Female | 2 | 0 |
| E14 | Male | Y | Y | Y | Homo | Male | 3 | 0 |
| E15 | Female |  | X | X | Het=Female | Female | 3 | 0 |
| E16 | Male |  |  |  | Homo | Unknown |  |  |
| E17 | Female |  | X | X | Homo | Female | 2 | 0 |
| E18 | Female |  | X | X | Het=Female | Female | 3 | 0 |
| E19 | Female |  | X | X | Het=Female | Female | 3 | 0 |
| E20 | Male |  |  |  |  | Unknown |  |  |
| E21 | Female |  | X | X |  | Female | 2 | 0 |
| E22 | Male | Y | Y | Y | Homo | Male | 3 | 0 |
| E23 | Female |  | X | X | Homo | Female | 2 | 0 |
| E24 | Male | Y | Y | Y | Homo | Male | 3 | 0 |
| E25 | Male | Y | Y | Y | Homo | Male | 3 | 0 |
| E26 | Male | Y |  |  | Homo | Male | 1 | 0 |
| E27 | Female |  | X | X | Het=Female | Female | 3 | 0 |
| E28 | Male | Y | Y | Y | Homo | Male | 3 | 0 |
| E29 | Female |  | X | X | Het=Female | Female | 3 | 0 |
| E30 | Female |  | X | X | Het=Female | Female | 3 | 0 |
| E31 | Female |  | X | X | Het=Female | Female | 3 | 0 |
| E32 | Male | Y | Y | Y | Homo | Male | 3 | 0 |
| E33 | Female |  | X | X | Het=Female | Female | 3 | 0 |
| E34 | Male |  | Y | Y | Homo | Male | 2 | 0 |

No sexing results disagreed when comparing with the sex based on morphology and any DNA sex-typing marker result. Only a proportion of markers detect each sex (3 detect males, 2 markers detect females and C11 detects approximately 80% of females). Of the 4 markers, one only amplifies in males (COO) and another marker only detects females (C11) . To be confident we only assigned sex if a minimum of 2 markers amplified. 29 individuals (85%) agree with morphometric sex for a minimum of 2 sex markers.

**Full data set**

Methods

163 samples (faecal, hair and tissue) were collected from both *Camelus bactrianus* and *Camelus ferus* in Mongolia and China (see main text). DNA was extracted, depending on sample type, using either Qiagen DNeasy blood and tissue kits (Tissue, blood or hair) or the Qiamp fast DNA Stool Mini kit (faeces). Extraction controls were included in each batch of extractions, replacing samples with ddH2O. Extracted DNA was used to amplify the 4 sex linked markers using the multitube approach[32], with each PCR repeated 3 times and a negative control (ddH20) included in each plate. 3ul volume PCRs were performed with the following:: 1 μl DNA, 1 μl of primer mix (23 μl Low TE ( 10mM Tris-HCl (pH 8.0) and 0.1mM EDTA (pH 8.0).), 1 μl forward and 1 μl reverse primers (both at 0.2? uM) and 1 μl QIAGEN Multiplex PCR Master Mix (supplied with the QIAGEN Multiplex PCR Kit, Cat. No. / ID: 206145). The PCR was incubated at 95 degrees for 15 min, 35 cycles of 95 degrees for 30 seconds, 60 degrees for 30 seconds, 72 degrees for 40 seconds. Incubate at 72 degrees for 4 mins. Genotypes were scored using the Genemapper software. Each sample was tested with each marker 3 times. For that sample to be scored it must show at least 2 matching repeats and no mismatches.

COO- a 170 bp amplicon indicates the presence of a Y chromosome, indicating the individual is male (the X chromosome does not amplify)

CTJ- shows a 146 bp amplicon in the presence of an X chromosome, indicating the individual is female and shows a 144 bp amplicon in the presence of an Y chromosome, indicating the individual is male.

CJE- shows a 181 bp amplicon in the presence of an X chromosome, indicating the individual is female and shows a 178 bp amplicon in the presence of an Y chromosome, indicating the individual is male.

C11- is X-linked (Y chromosome doesn’t not amplify) and so considered female (XX) if heterozygote. Males (XY) are always homozygotes and cannot be heterozygotes since males have only one X chromosome. Females can also be homozygotes, but any heterozygotes must be female for marker C11. Homozygotes were not sexed, since homozygotes could be female or male for this marker. All heterozygotes were scored as female.

Results:

Table 7: Results from marker testing across full available samples (N=163). Samples include Wild camel *Camelus ferus*, Bactrian camels *Camelus bactrianus*, and hybrids. Samples are a mixture of faecal, hair and tissue.

| ID | Sample type | COO_Y | CTJ_Y | CJE_Y | C11_X | Sex | No markers that agree | No markers that disagree |
| --- | --- | --- | --- | --- | --- | --- | --- | --- |
| *Camelus bactrianus* | | | | | | | | |
| Khara | Blood |  | X |  | HOM | F | 3 | 0 |
| B1 | Faecal |  |  |  |  | U |  |  |
| B2 | Faecal |  | X |  | HET- FEMALE | F | 3 | 0 |
| B3 | Faecal |  | X |  | HOM | F | 2 | 0 |
| B4 | Faecal |  | X |  | HET- FEMALE | F | 3 | 0 |
| B5 | Faecal |  | X |  | HOM | F | 2 | 0 |
| Hybrid- *Camelus ferus x Camelus bactrianus* | | | | | | | | |
| AWC122 | Faecal |  | X |  | HET- FEMALE | F | 3 | 0 |
| AWC132 | Faecal |  |  |  |  | U |  |  |
| AWC134 | Faecal |  |  |  | HOM | U |  |  |
| AWC179 | Faecal | Y |  | Y |  | M | 2 | 0 |
| AWC248 | Faecal | Y | Y | Y | HOM | M | 4 | 0 |
| AWC272 | Faecal | Y | Y | Y | HOM | M | 4 | 0 |
| AWC278 | Faecal | Y | Y | Y | HOM | M | 4 | 0 |
| AWC279 | Faecal |  | Y | Y | HOM | M | 3 | 0 |
| AWC282 | Faecal | Y | Y | Y |  | M | 3 | 0 |
| AWC299 | Faecal | Y | Y | Y | HOM | M | 4 | 0 |
| AWC300 | Faecal |  | X |  | HET- FEMALE | F | 3 | 0 |
| AWC305 | Faecal | Y | Y | Y | HOM | M | 4 | 0 |
| AWC308 | Faecal |  | X |  | HET- FEMALE | F | 3 | 0 |
| AWC310 | Faecal |  | X |  | HET- FEMALE | F | 3 | 0 |
| AWC314 | Faecal | Y | Y | Y | HOM | M | 4 | 0 |
| AWC327 | Faecal | Y | Y | Y | HOM | M | 4 | 0 |
| AWC328 | Faecal |  | X |  | HOM | F | 2 | 0 |
| AWC33 | Faecal |  | Y |  |  | M | 1 | 0 |
| AWC330 | Faecal | Y | Y | Y | HOM | M | 4 | 0 |
| AWC331 | Faecal | Y | Y | Y | HOM | M | 4 | 0 |
| AWC334 | Faecal |  | X |  | HOM | F | 2 | 0 |
| AWC335 | Tissue | Y | Y | Y | HOM | M | 4 | 0 |
| AWC336 | Faecal | Y |  | Y | HOM | M | 3 | 0 |
| AWC345 | Tissue? |  | X |  | HOM | F | 2 | 0 |
| AWC35 | Faecal |  | X |  |  | F | 2 | 0 |
| AWC38 | Faecal |  | X |  |  | F | 2 | 0 |
| AWC50 | Faecal |  |  |  |  | U |  |  |
| AWC55 | Faecal | Y |  | Y | HOM | M | 3 | 0 |
| AWC64 | Faecal |  | X |  | HET- FEMALE | F | 3 | 0 |
| AWC73 | Faecal | Y | Y | Y | HOM | M | 4 | 0 |
| AWC80 | Faecal |  | X |  | HOM | F | 2 | 0 |
| CHYB14 | Hair |  | X |  | HOM | F | 2 | 0 |
| CHYB33 | Hair | Y | Y | Y | HOM | M | 4 | 0 |
| CHYB34 | Hair | Y | Y | Y | HOM | M | 4 | 0 |
| CHYB36 | Hair | Y | Y | Y | HOM | M | 4 | 0 |
| CHYB37 | Hair |  | X |  | HET- FEMALE | F | 3 | 0 |
| CHYB38 | Hair | Y | Y | Y | HOM | M | 4 | 0 |
| CHYB39 | Hair |  | X |  | HET- FEMALE | F | 3 | 0 |
| CHYB40 | Hair |  | X |  | HET- FEMALE | F | 3 | 0 |
| CHYB41 | Hair | Y | Y | Y | HOM | M | 4 | 0 |
| CHYB42 | Hair |  | X | X | HET- FEMALE | F | 4 | 0 |
| CHYB43 | Hair |  | X |  | HET- FEMALE | F | 3 | 0 |
| CHYB44 | Hair | Y | Y | Y | HOM | M | 4 | 0 |
| CHYB45 | Hair |  | X |  | HOM | F | 2 | 0 |
| CHYB46 | Hair |  | X |  | HOM | F | 2 | 0 |
| CHYB47 | Hair |  |  |  | HET- FEMALE | F | 2 | 0 |
| CHYB48 | Hair |  | X | X | HOM | F | 2 | 0 |
| CHYB53 | Hair |  | X |  | HET- FEMALE | F | 3 | 0 |
| CWC2 | Hair | Y | Y | Y | HOM | M | 4 | 0 |
| CWC9 | Hair | Y | Y | Y |  | M | 3 | 0 |
| HYB17 | Blood | Y |  |  | HOM | M | 2 | 0 |
| HYB57 | Hair | Y | Y | Y | HOM | M | 4 | 0 |
| wc117 | Hair | Y | Y | Y | HOM | M | 4 | 0 |
| WC119 | Hair | Y | Y | Y | HOM | M | 4 | 0 |
| WC121 | Hair | Y | Y | Y | HOM | M | 4 | 0 |
| WC205 | Hair | Y | Y | Y | HOM | M | 4 | 0 |
| WC207 | Tissue |  | Y |  |  | M | 1 | 0 |
| WC208 | Hair | Y |  |  | HOM | M | 2 | 0 |
| WC210 | Tissue | Y | Y | Y | HOM | M | 4 | 0 |
| WC225 | Hair |  |  |  |  | U |  |  |
| WC242 | Hair | Y | Y | Y | HOM | M | 4 | 0 |
| WC246 | Hair | Y | Y | Y |  | M | 3 | 0 |
| WC249 | Hair |  | Y | Y |  | M | 2 | 0 |
| WC251 | Hair |  | Y |  | HOM | M | 2 | 0 |
| *Camelus ferus* | | | | | | | | |
| AWC11 | Faecal | Y | Y | Y | HOM | M | 4 | 0 |
| AWC111 | Faecal |  | Y | Y | HOM | M | 3 | 0 |
| AWC112 | Faecal |  | Y |  | HOM | M | 2 | 0 |
| AWC113 | Faecal |  |  |  | HET- FEMALE | F | 2 | 0 |
| AWC114 | Faecal | Y | Y | Y | HOM | M | 4 | 0 |
| AWC115 | Faecal | Y | Y | Y | HOM | M | 4 | 0 |
| AWC118 | Faecal | Y | Y | Y | HOM | M | 4 | 0 |
| AWC119 | Faecal | Y | Y | Y |  | M | 3 | 0 |
| AWC120 | Faecal |  | X |  | HET- FEMALE | F | 3 | 0 |
| AWC123 | Faecal |  |  |  | HOM | U |  |  |
| AWC124 | Faecal | Y | Y | Y | HOM | M | 4 | 0 |
| AWC125 | Faecal |  | X |  | HET- FEMALE | F | 3 | 0 |
| AWC127 | Faecal | Y | Y | Y | HOM | M | 4 | 0 |
| AWC128 | Faecal |  | X |  | HET- FEMALE | F | 3 | 0 |
| AWC129 | Faecal |  |  |  | HET- FEMALE | F | 2 | 0 |
| AWC13 | Faecal |  |  |  |  | U |  |  |
| AWC130 | Faecal |  | Y | Y | HOM | M | 3 | 0 |
| AWC131 | Faecal | Y | Y | Y | HOM | M | 4 | 0 |
| AWC133 | Faecal |  | X |  | HOM | F | 3 | 0 |
| AWC135 | Faecal |  | X |  | HET- FEMALE | F | 3 | 0 |
| AWC136 | Faecal | Y | Y | Y | HOM | M | 4 | 0 |
| AWC156 | Hair |  |  |  |  | U |  |  |
| AWC166 | Hair |  |  |  |  | U |  |  |
| AWC184 | Faecal |  | X |  | HOM | F | 2 | 0 |
| AWC20 | Faecal |  | X |  |  | F | 2 | 0 |
| AWC200 | Faecal | Y | Y | Y | HOM | M | 4 | 0 |
| AWC21 | Faecal |  |  |  | HET- FEMALE | F | 2 | 0 |
| AWC215 | Faecal |  | X |  | HOM | F | 2 | 0 |
| AWC22 | Faecal |  | X |  | HOM | F | 2 | 0 |
| AWC243 | Faecal | Y | Y | Y | HOM | M | 4 | 0 |
| AWC245 | Faecal |  | Y |  | HOM | M | 2 | 0 |
| AWC252 | Faecal |  |  |  | HET- FEMALE | F | 2 | 0 |
| AWC256 | Faecal | Y | Y | Y | HOM | M | 4 | 0 |
| AWC27 | Faecal |  | Y |  |  | M | 1 | 0 |
| AWC275 | Faecal |  | X |  | HOM | F | 2 | 0 |
| AWC283 | Faecal | Y |  | Y | HOM | M | 3 | 0 |
| AWC286 | Faecal |  | X |  | HET- FEMALE | F | 3 | 0 |
| AWC289 | Faecal |  | Y | Y | HOM | M | 3 | 0 |
| AWC29 | Faecal |  |  |  |  | U |  |  |
| AWC294 | Faecal |  | X |  | HOM | F | 2 | 0 |
| AWC298 | Faecal |  | X |  | HOM | F | 2 | 0 |
| AWC302 | Faecal | Y | Y | Y | HOM | M | 4 | 0 |
| AWC303 | Faecal |  | X |  | HET- FEMALE | F | 3 | 0 |
| AWC306 | Faecal |  | X |  | HOM | F | 2 | 0 |
| AWC309 | Faecal |  | X |  | HET- FEMALE | F | 3 | 0 |
| AWC311 | Faecal | Y | Y | Y | HOM | M | 4 | 0 |
| AWC317 | Faecal | Y | Y | Y | HOM | M | 4 | 0 |
| AWC319 | Faecal |  |  |  | HET- FEMALE | F | 2 | 0 |
| AWC32 | Faecal |  | X |  |  | F | 2 | 0 |
| AWC329 | Faecal | Y | Y | Y | HOM | M | 4 | 0 |
| AWC332 | Faecal | Y | Y | Y | HOM | M | 4 | 0 |
| AWC333 | Faecal | Y | Y | Y | HOM | M | 4 | 0 |
| AWC337 | tissue | Y | Y | Y | HOM | M | 4 | 0 |
| AWC34 | Faecal |  |  |  |  | U |  |  |
| AWC341 | tissue |  | Y |  | HOM | M | 2 | 0 |
| AWC347 | Faecal | Y | Y | Y | HOM | M | 4 | 0 |
| AWC36 | Faecal | Y | Y | Y | HOM | M | 4 | 0 |
| AWC37 | Faecal |  | X |  |  | F | 2 | 0 |
| AWC49 | Faecal |  | X |  | HET- FEMALE | F | 3 | 0 |
| AWC61 | Faecal | Y | Y | Y | HOM | M | 4 | 0 |
| AWC63 | Faecal |  | X |  | HOM | F | 2 | 0 |
| AWC65 | Hair |  |  |  | HOM | U |  |  |
| AWC67 | Faecal | Y | Y | Y | HOM | M | 4 | 0 |
| AWC68 | Faecal |  | Y |  |  | M | 1 | 0 |
| AWC70 | Faecal |  |  |  | HET- FEMALE | F | 2 | 0 |
| AWC71 | Faecal | Y | Y | Y | HOM | M | 4 | 0 |
| AWC72 | Faecal |  |  |  |  | U |  |  |
| AWC74 | Faecal |  |  |  |  | U |  |  |
| AWC82 | Faecal | Y | Y | Y | HOM | M | 4 | 0 |
| AWC86 | Faecal |  | X |  | HOM | F | 2 | 0 |
| CWC5 | Hair | Y | Y | Y | HOM | M | 4 | 0 |
| R120 | Faecal |  |  |  |  | U |  |  |
| SK | tissue |  | X |  | HET- FEMALE | F | 3 | 0 |
| WC115 | Hair | Y | Y | Y | HOM | M | 4 | 0 |
| WC118 | Hair |  | X |  | HOM | F | 2 | 0 |
| WC120 | Hair | Y | Y | Y | HOM | M | 4 | 0 |
| WC18 | Blood |  | Y | Y | HOM | M | 3 | 0 |
| wc20 | Blood |  | X |  | HOM | F | 3 | 0 |
| WC202 | Hair |  | X |  | HET- FEMALE | F | 3 | 0 |
| WC221 | Hair |  | X |  | HOM | F | 2 | 0 |
| WC243 | Hair | Y | Y | Y | HOM | M | 4 | 0 |
| WC254 | Hair |  | Y | Y | HOM | M | 3 | 0 |
| WC257 | Blood | Y | Y | Y |  | M | 3 | 0 |
| WC261 | tissue |  | X | X | HET- FEMALE | F | 4 | 0 |
| WC264 | Hair |  | Y |  | HOM | M | 2 | 0 |
| WC31 | tissue |  | X |  |  | F | 2 | 0 |
| WC41 | tissue | Y | Y | Y | HOM | M | 4 | 0 |
| Disagree | | | | | | | | |
| WC162 | Hair | Y | Y | Y | HET- FEMALE | M/U | 3 | 1 |
| AWC117 | Faecal | Y | Y | Y | HET- FEMALE | M/U | 3 | 1 |
| WC164 | Hair | Y | Y | Y | HET- FEMALE | M/U | 3 | 1 |
| AWC209 | Faecal | Y | X |  | HOM | U | 1 | 1 |
| AWC110 | Faecal |  | X | Y | HET- FEMALE | F/U | 3 | 1 |
| WC165 | Hair | Y | Y | Y | HET- FEMALE | M/U | 3 | 1 |

**Summary:**

- Of the 163 samples, 142 (87%) were successfully scored to suggest a sex (minimum 1 marker with 2 successful repeats in each, with no markers disagreeing).
- Of those 142, 61 (42%) are female and 81 (57%) are males
- 15 were not possible to score as they did not amplify to the required minimum repeats. 6 had disagreeing scores between markers, which may be a result of what contamination during the PCR process - so 21 (13%) cannot be used to determine sex of that sample.
- If we consider sexing with a minimum of 2 agreeing markers then 94 (57%) samples can be sexed. Of the 47 that were scored in only one marker, 36 (76%) are female.
- Considering those samples only from the GGASPA (N=115) 45 are female (39%) and 70 are male (61%). This result, showing more male than female in the GGASPA, is probably an artifact of poor amplification of the polymorphic markers in poor quality samples. When used on DNA extracted from tissue samples in known sex individuals of the captive herd this sex discrepancy is not seen. In the 163 samples, the 21 samples that didn’t amplify were all either faecal or hair. All 15 blood and tissue samples amplified. 14% of the faecal samples didn’t amplify and 15% of the hair samples didn’t amplify. Of the 6 samples that disagreed, 3 were faecal and 3 were hair. Faecal (63%) and hair (28%) samples make up the majority of the sample set (91 %) (Table 4):

|  | No. Samples | Number with no amplification | % with no amplification |
| --- | --- | --- | --- |
| Blood | 5 | 0 | 0 |
| Faecal | 103 | 14 | 14 |
| Hair | 45 | 7 | 15 |
| Tissue | 10 | 0 | 0 |

Table 4: Sample types (blood, faeces, hair and tissue) with number and percentage that did not amplify.

**Sex ratio demographics**

To determine the sex ratio in the wild population we used only the 2 markers with fixed size X and fixed size Y homolog that differ in size: CJE and CTJ. This allows a comparison as both markers can determine either male or female, whereas COO and C11 determine only male (COO) or female (C11).

| Population | Sex determined (%) | Sex determined using both CJE/CJT (%) | Sex determined using either CJE/CJT (%) | Overall % Male | Overall % Female |
| --- | --- | --- | --- | --- | --- |
| Captive herd N=34 | 88 | 81 | 6 | 37 | 63 |
| Full available sample set  N=163 | 81 | 42 | 39 | 59 | 41 |

Table 5: Sex determined in each population in either one or both polymorphic markers. In the captive population, 30 of the 34 were scored with at least one sex marker (CJE or CTJ). 28 were scored in both- 18 female (64%), 10 male (36%) and 2 were scored in only sex marker. In the full available data set 133 out of the 163 were scored in either one or both markers. 69 were scored in both CJE and CTJ, 3 female (4%), 66 male (96%) and 64 in only sex marker- 51 female (80%), 13 male (20%). In the captive herd, overall, 19 were scored as female (63%) 11 scored as male (37%)- which is true when compared to known sex. When looking at the full available data set overall 41% female, 59% male. Considering only those samples from the GGASPA N=107, 38 are female 36%, 69 are male 64%
