# Supplementary material for: Challenges of Introgression in Conservation: Genetic Diversity of the Endangered Wild Camel (Camelus ferus) in Mongolia
Source: Ecol Evol. 2026 Mar 29;16(4):e73293. doi: 10.1002/ece3.73293 (PMC13107281; doi:10.1002/ece3.73293)
Supplement: Supplementary file 6 — Appendix S7: ece373293‐sup‐0006‐AppendicesS7‐S10.docx. Appendix S8: ece373293‐sup‐0006‐AppendicesS7‐S10.docx. Appendix S9: ece373293‐sup‐0006‐AppendicesS7‐S10.docx. Appendix S10: ece373293‐sup‐0006‐AppendicesS7‐S10.docx. [file ECE3-16-e73293-s005.docx]

**Annex 7- Null Alleles**

Table 8: Micro-checker, Null allele check on all 257 samples, split into populations determined using q=0.5. Includes all data. No loci show evidence for a null allele. This population is probably in Hardy Weinberg equilibrium.

| **Locus** | **Null Present** | **Oosterhout** | **Chakraborty** | **Brookfield 1** | **Brookfield 2** |
| --- | --- | --- | --- | --- | --- |
| KS01 | no | -0.0799 | -0.0631 | -0.0498 | 0 |
| KS02 | no | 0 | 0 | 0 | 0 |
| KS03 | no | 0 | 0 | 0 | 0.1543 |
| KS04 | no | 0 | 0 | 0 | 0 |
| KS05 | no | -0.0899 | -0.0786 | -0.0553 | 0.0785 |
| KS06 | no | -0.093 | -0.087 | -0.0635 | 0 |
| KS07 | no | -0.1751 | -0.1194 | -0.1073 | 0 |
| KS08 | no | -0.0373 | -0.0186 | -0.0025 | 0.1453 |
| KS09 | no | 0.0642 | 0.0847 | 0.0258 | 0.1563 |
| YWLL36 | no | -0.0585 | -0.0672 | -0.0408 | 0 |
| CVRL07 | no | 0.0114 | 0.0168 | 0.0107 | 0.2057 |
| LCA65 | no | -0.1242 | -0.052 | -0.0194 | 0 |
| VOLP08 | no | -0.0986 | -0.0741 | -0.0523 | 0.3854 |
| VOLP10 | no | 0.1022 | 0.1327 | 0.0856 | 0.3977 |
| VOLP32 | no | -0.0123 | -0.0173 | -0.0111 | 0 |
| VOLP59 | no | -0.0767 | -0.0687 | -0.0498 | 0.0776 |

**Annex 8- AMOVA**

Analysis of molecular variance AMOVA showed that the greatest variance was within individuals (75%), then among populations (21%) and finally among individuals (1%) (Table 1). This reflects the overlap between all these designated populations. As expected, the largest FST values are between the two species *C. bactrianus* and the captive population (FST=0.397), with wild and Bactrian showing similar (FST=0.372). Hybrids show higher FST when compared to Bactrian (FST=0.165) than wild (FST=0.074) or captive (FST=0.105).

Table 9: Summary AMOVA table with degrees of freedom (df), Sum of squared deviation (SS), Mean sum of squares (MS), Estimated Variation (Est.Var) and percentage variance (%).

| **Source** | **df** | **SS** | **MS** | **Est. Var.** | **%** |
| --- | --- | --- | --- | --- | --- |
| **Among Populations** | 3 | 394.857 | 131.619 | 1.039 | 21% |
| **Among Individuals** | 253 | 1040.930 | 4.114 | 0.217 | 4% |
| **Within Individuals** | 257 | 945.736 | 3.680 | 3.680 | 75% |
| **Total** | 513 | 2381.522 |  | 4.936 | 100% |

Table 10- Pairwise FST Analysis. FST Values below the diagonal, p values above.

|  | **C. ferus** | **C. bactrianus** | **Hybrids** | **Ex-situ** |
| --- | --- | --- | --- | --- |
| **C. ferus** | 0.000 | 0.001 | 0.001 | 0.001 |
| **C. bactrianus** | 0.372 | 0.000 | 0.001 | 0.001 |
| **Hybrids** | 0.074 | 0.165 | 0.000 | 0.001 |
| **Ex-situ** | 0.043 | 0.397 | 0.105 | 0.000 |

**Annex 9: Alleles per locus**

Table 11- Alleles per locus

|  | **Bactrian (55)** | **Captive (45)** | **Hybrid (41)** | **Wild (116)** |
| --- | --- | --- | --- | --- |
| KS01 | 7 | 4 | 7 | 7 |
| KS02 | 9 | 2 | 8 | 6 |
| KS03 | 12 | 1 | 7 | 8 |
| KS04 | 3 | 1 | 4 | 2 |
| KS05 | 5 | 3 | 6 | 4 |
| KS06 | 4 | 4 | 6 | 4 |
| KS07 | 3 | 4 | 4 | 5 |
| KS08 | 7 | 2 | 7 | 7 |
| KS09 | 4 | 3 | 5 | 4 |
| YWLL36 | 5 | 4 | 5 | 6 |
| CVRL07 | 3 | 3 | 5 | 4 |
| LCA65 | 5 | 5 | 6 | 4 |
| VOLP08 | 5 | 3 | 7 | 5 |
| VOLP10 | 8 | 3 | 11 | 8 |
| VOLP32 | 2 | 3 | 3 | 3 |
| VOLP59 | 2 | 3 | 4 | 9 |
| Total | 84 | 48 | 95 | 86 |
| Mean | 9.882352941 | 5.647058824 | 11.17647059 | 10.11764706 |
| Median | 5 | 3 | 6 | 5 |
| Min | 2 | 1 | 3 | 2 |
| max | 12 | 5 | 11 | 9 |

**Annex 10- mtDNA Results**


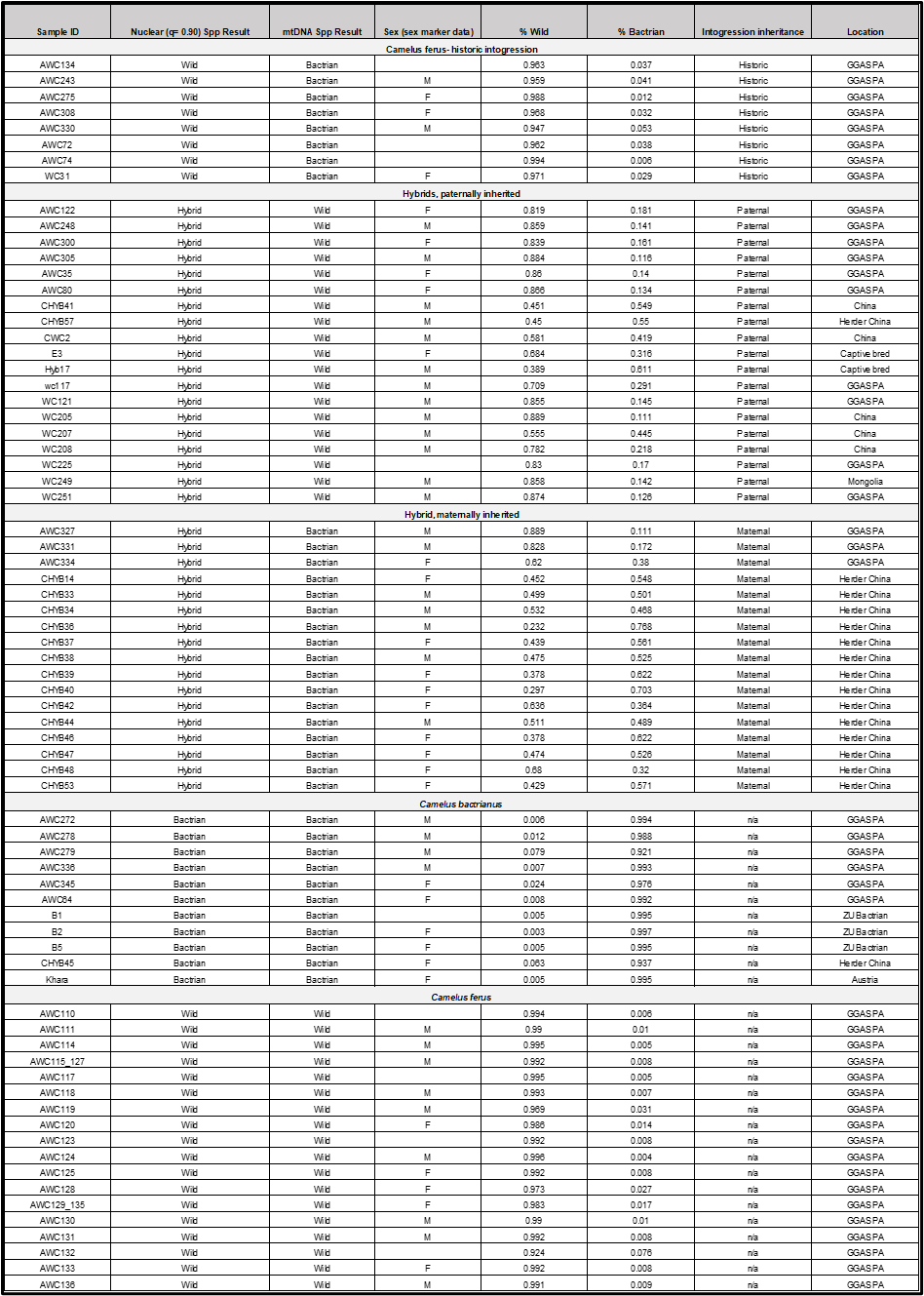


**Table** 5: MtDNA results showing all available data for *C. bactrianus*, hybrids and historic introgressed *C. ferus*, plus example selection of *C. ferus* individual AWC110-136. Species determined by qi=0.90. Nuclear species result determined my microsatellite testing (qi=0.90), mitochondrial DNA species result determined by mitochondrial PCR-RFLP or sequencing analysis, Sex determined by the sex-linked markers and introgression inheritance being the direction of inheritance presumed from maternal mtDNA.
