# Supplementary material for: Challenges of Introgression in Conservation: Genetic Diversity of the Endangered Wild Camel (Camelus ferus) in Mongolia
Source: Ecol Evol. 2026 Mar 29;16(4):e73293. doi: 10.1002/ece3.73293 (PMC13107281; doi:10.1002/ece3.73293)
Supplement: Supplementary file 7 — Appendix S11: ece373293‐sup‐0007‐AppendixS11.docx. [file ECE3-16-e73293-s004.docx]

**Annex 11: Additional Structure Analysis.**


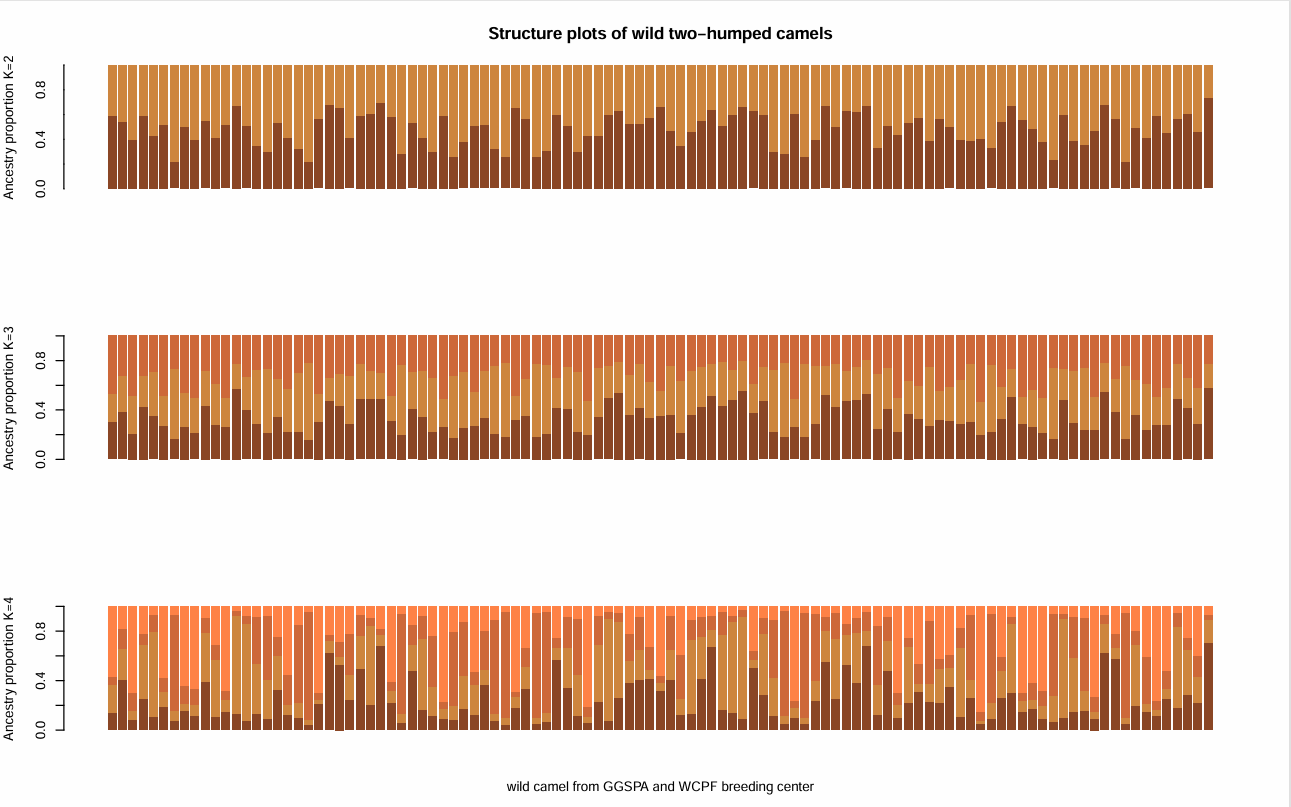


Figure 1: Structure plots from Structure Harvester for K=2, K=3 and K=4.


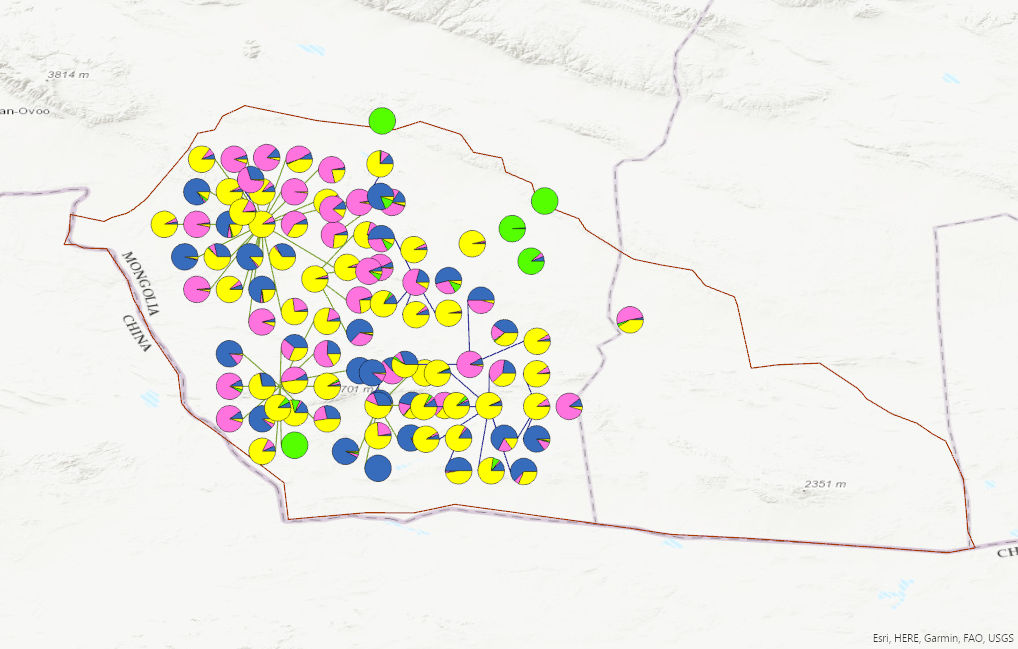


Figure 2: K=4 visualised across the GGASPA.


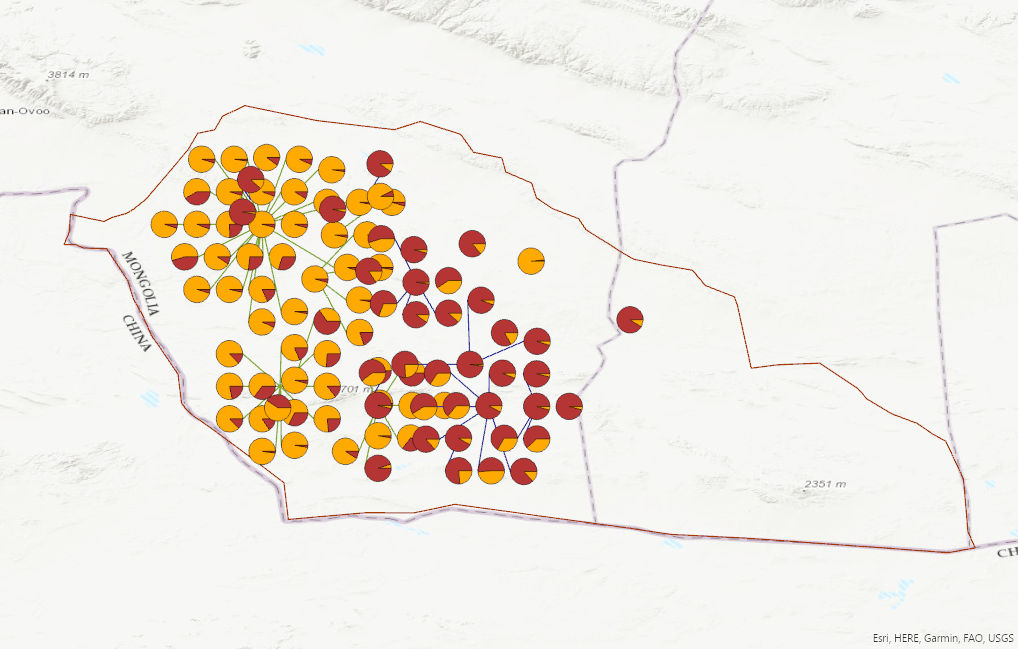


Figure 3: K=2 with pure *C. bactrianus* removed from analysis. Visualised across the GGASPA.


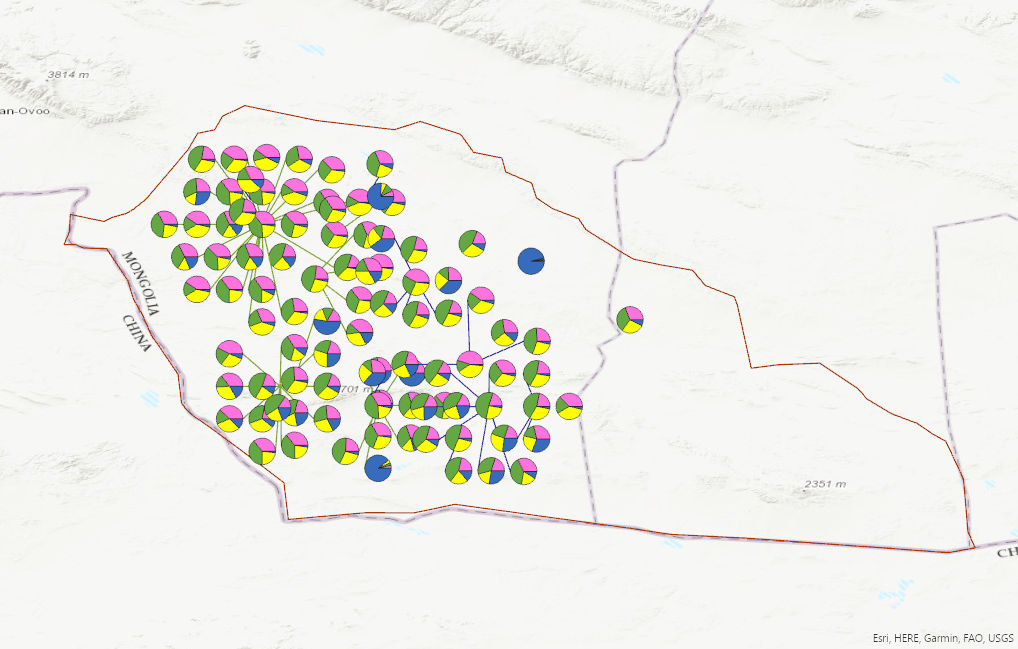


Figure 4: K=4 with pure *C. bactrianus* removed from analysis. Visualised across the GGASPA.


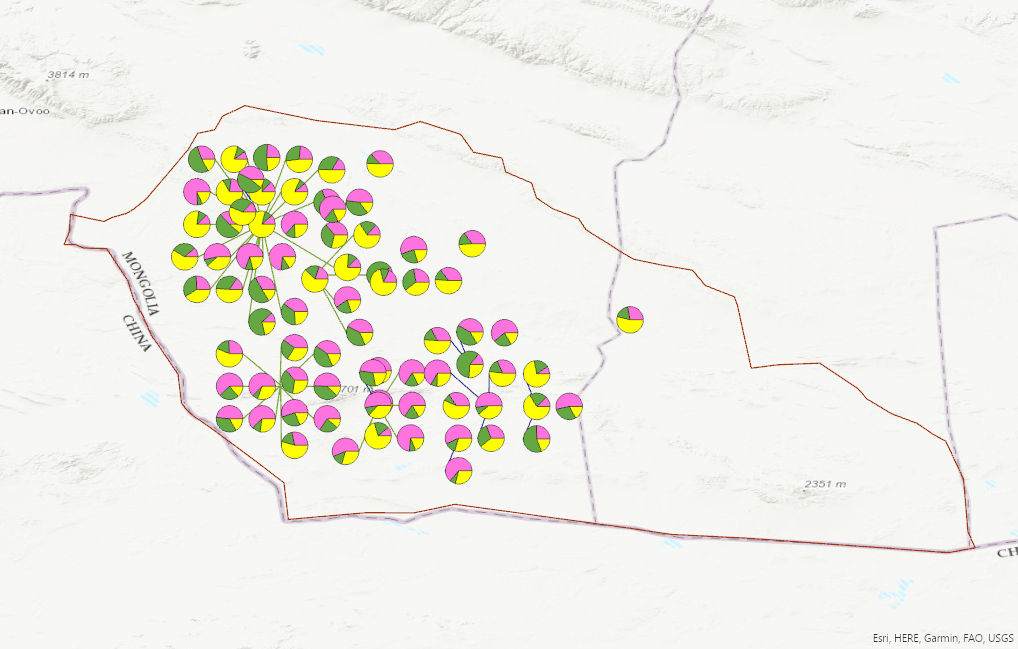


Figure 5: K=3 with pure *C. bactrianus* and hybrids removed from analysis. Visualised across the GGASPA.
